# Chaperone addiction of toxin–antitoxin systems

**DOI:** 10.1038/ncomms13339

**Published:** 2016-11-09

**Authors:** Patricia Bordes, Ambre Julie Sala, Sara Ayala, Pauline Texier, Nawel Slama, Anne-Marie Cirinesi, Valérie Guillet, Lionel Mourey, Pierre Genevaux

**Affiliations:** 1 Laboratoire de Microbiologie et de Génétique Moléculaires, Centre de Biologie Intégrative (CBI), Université de Toulouse, CNRS, UPS, 31062 Toulouse, France; 2 Institut de Pharmacologie et de Biologie Structurale, Université de Toulouse, CNRS, UPS, 31077 Toulouse, France; 3 Present address: Department of Molecular Biosciences, Northwestern University, Evanston, Illinois 60208, USA

## Abstract

Bacterial toxin–antitoxin (TA) systems, in which a labile antitoxin binds and inhibits the toxin, can promote adaptation and persistence by modulating bacterial growth in response to stress. Some atypical TA systems, known as tripartite toxin–antitoxin–chaperone (TAC) modules, include a molecular chaperone that facilitates folding and protects the antitoxin from degradation. Here we use a TAC module from *Mycobacterium tuberculosis* as a model to investigate the molecular mechanisms by which classical TAs can become ‘chaperone-addicted'. The chaperone specifically binds the antitoxin at a short carboxy-terminal sequence (chaperone addiction sequence, ChAD) that is not present in chaperone-independent antitoxins. In the absence of chaperone, the ChAD sequence destabilizes the antitoxin, thus preventing toxin inhibition. Chaperone–ChAD pairs can be transferred to classical TA systems or to unrelated proteins and render them chaperone-dependent. This mechanism might be used to optimize the expression and folding of heterologous proteins in bacterial hosts for biotechnological or medical purposes.

## Abstract (web summary)

Some bacterial toxin-antitoxin systems consist of a labile antitoxin that inhibits a toxin, and a chaperone that stabilizes the antitoxin. Here, Bordes *et al*. identify a sequence within the antitoxin to which the chaperone binds and which can be transferred to other proteins to make them chaperone-dependent.

## Graphical abstract

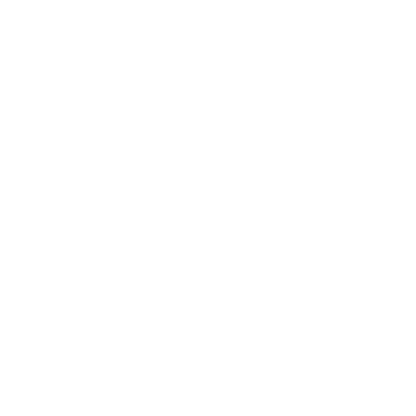

Bacterial type II toxin–antitoxin (TA) systems are small genetic modules typically composed of a toxin and a more labile cognate antitoxin, which binds and inhibits the toxin^1^,^2^,^3^,^4^,^5^. In response to certain stress, the antitoxin is rapidly degraded by proteases and the free active toxin generally targets essential cellular processes, leading to a reversible growth inhibition known to facilitate bacterial persistence to drugs, virulence and survival in response to environmental insults^2^,^3^,^4^,^5^,^6^,^7^,^8^,^9^.

Tripartite toxin–antitoxin–chaperone (TAC) systems are atypical TA modules composed of a two-component type II TA, generally belonging to the well-conserved HigBA, HicAB or MqsRA TA families, and a molecular chaperone homologous to the export chaperone SecB, located on the same operon^10^,^11^. To date, the TAC system of the major human pathogen *Mycobacterium tuberculosis* is the best characterized^4^,^10^,^12^. It is encoded by three genes organized in an operon, *Rv1955-Rv1956-Rv1957*, respectively, encoding the TA pair (*Mtb*-HigB1 and *Mtb-*HigA1) and the *Mtb*-SecB^TA^ chaperone^10^,^12^. Transcription of *TAC* is induced in host phagocytes and by several stresses relevant for *M. tuberculosis*, including DNA damage, heat shock, nutrient starvation, hypoxia and multidrug persistence^10^,^13^. In addition, ectopic expression of the *Mtb*-HigB1 toxin severely inhibits *M. tuberculosis*, *M. marinum*, *M. smegmatis* and *Escherichia coli* growth^13^,^14^,^15^, and affects the relative abundance of over 30 transcripts in *M. tuberculosis*^15^.

Previous work demonstrated that *Mtb*-HigBA1 forms a *bona fide* TA that is specifically controlled by *Mtb*-SecB^TA^ through a direct interaction between the chaperone and the antitoxin, which allows antitoxin folding and protection from degradation, and the subsequent inhibition of the toxin^12^. *Mtb*-SecB^TA^ was shown to functionally replace the canonical *E. coli* SecB chaperone during protein export *in vivo*, thus suggesting that *M. tuberculosis* could make use of such export chaperone function under certain conditions. Whether the activation of TAC is directly coupled with protein export is not known^12^.

In this work, we have used the TAC system of *M. tuberculosis* as a model to shed light on the molecular mechanism that renders TA pairs chaperone-dependent. We show that TAC antitoxins possess a short chaperone addiction sequence, named ChAD, at their carboxy-terminal end, which efficiently prevents antitoxin folding and specifically recruits the SecB^TA^ chaperone. Grafting the ChAD sequence of *Mtb*-HigA1 to unrelated recombinant proteins or to chaperone-independent antitoxins of classical two-component TA systems renders them chaperone-dependent, thus indicating that chaperone addiction is transferable.

## Results

### The C terminus of TAC antitoxin confers chaperone addiction

**Figure 1:**
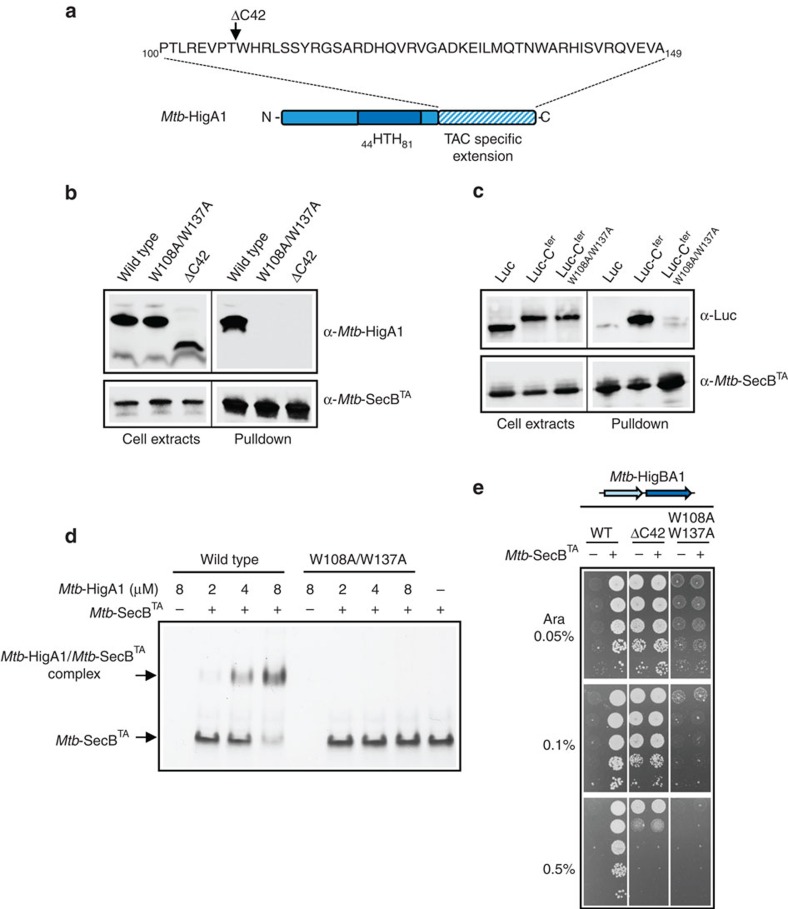
*Mtb*-SecB^TA^ interacts with the C-terminal region of *Mtb*-HigA1. (**a**) Schematic representation of *Mtb*-HigA1. The dark blue box represents the helix-turn-helix (HTH) motif and the blue hachures the TAC-specific C-terminal extension. The deletion in ΔC42 is indicated with an arrow. (**b**) *In vivo* interaction between *Mtb*-SecB^TA^ and *Mtb*-HigA1. *In vivo* pulldowns of His-tagged *Mtb*-SecB^TA^ and the different *Mtb*-HigA1 (wild type), *Mtb*-HigA1^W108A/W137A^ (W108A/W137A) and *Mtb*-HigA1^ΔC42^ (ΔC42) proteins were revealed using anti-*Mtb*-HigA1 or anti-*Mtb*-SecB^TA^ antibodies. (**c**) *In vivo* interaction between *Mtb*-SecB^TA^ and the C-terminal extension of *Mtb*-HigA1 (aa 104–149) wild type (C^ter^) or mutant (C^ter W108A/W137A^) fused with luciferase (Luc). *In vivo* pulldowns of His-tagged *Mtb*-SecB^TA^ and the pK6-Luc constructs were revealed with anti-luciferase or anti-*Mtb*-SecB^TA^ antibodies. Full blots for **b** and **c** are shown in Supplementary Fig. 6. (**d**) *In vitro* native PAGE separation of complexes between *Mtb*-HigA1 or *Mtb*-HigA1^W108A/W137A^, at 2, 4 and 8 μM and *Mtb*-SecB^TA^ (16 μM). Full gel for **d** is shown in Supplementary Fig. 7. (**e**) Suppression of *Mtb*-HigB1 toxicity by *Mtb*-HigA1 and *Mtb*-SecB^TA^ in *E. coli*. Strains W3110 Δ*secB* containing the plasmid pSE (−) or pSE-*Mtb*-SecB^TA^ (+; with *Mtb*-SecB^TA^ under control of Ptrc promoter) were transformed with pK6-based plasmids harbouring *Mtb*-HigA1, *Mtb*-HigA1^ΔC42^ or *Mtb*-HigA1^W108A/W137A^ under control of P_BAD_ promoter, grown to mid-log phase, serially diluted and spotted on LB–ampicillin–kanamycin agar plates without IPTG and with arabinose as indicated. Plates were incubated at 37 °C overnight.

Analysis of TAC systems in bacterial genomes revealed that SecB chaperones can be associated with well-distinct antitoxin families, including HigA and MqsA, and to a lesser extent HicB^10^. Multiple alignments of TAC antitoxin sequences with their classical two-component antitoxins homologues indicate that in spite of relatively well-conserved antitoxin domains, mainly within DNA-binding regions, putative TAC antitoxins carry a carboxy-terminal extension, which is highly variable both in length and amino-acid composition (Supplementary Fig. 1). The C-terminal extension of *Mtb*-HigA1 likely consists of the last 45 amino acids and possesses two similar stretches of amino-acid residues (_108_WHRLSSYR_115_ and _137_WARHISVR_144_; Fig. 1a) that resemble *E. coli* SecB-binding motifs identified by peptide-binding scan of protein substrates; that is, short segments enriched in aromatic and basic residues, with acidic residues strongly disfavoured^16^. Therefore, we first asked whether the C-terminal extension of the *Mtb*-HigA1 TAC antitoxin was involved in *Mtb*-SecB^TA^ binding. Two *Mtb*-HigA1 mutants were engineered: the *Mtb*-HigA1^ΔC42^ deleted of its last 42 residues and the *Mtb*-HigA1^W108A/W137A^ mutant containing both W108A and W137A amino-acid substitutions (Fig. 1a). The mutants were tested for *Mtb*-SecB^TA^ binding by *in vivo* pulldown with co-expressed antitoxin and His-tagged chaperone. Remarkably, both mutations abolish interaction with the chaperone, revealing a key role for the C-terminal region of the TAC antitoxin in recruiting *Mtb*-SecB^TA^ (Fig. 1b). To test whether this region was sufficient to efficiently recruit *Mtb*-SecB^TA^, the segment encompassing amino acids 104–149 of *Mtb*-HigA1 was grafted to an unrelated model protein, namely, luciferase from *Photinus pyralis*, and the resulting chimera was tested for *in vivo* interaction with the chaperone, using pull-down experiments with co-expressed luciferase derivatives and the His-tagged chaperone^12^. The results presented in Fig. 1c clearly show that the C-terminal extension can indeed reroute the *Mtb*-SecB^TA^ chaperone towards luciferase. As observed for the antitoxin, the double W108A/W137A mutation in the grafted C-terminal extension also suppressed the interaction. To further confirm the role of the C-terminal extension in chaperone binding, both *Mtb*-HigA1 and *Mtb*-HigA1^W108A/W137A^ proteins were purified and tested for their ability to form a stable complex with *Mtb*-SecB^TA^ *in vitro* in a native gel separation assay. As expected, incubation of *Mtb*-HigA1 wild type (WT) with the chaperone leads to the formation of a higher-molecular-weight *Mtb*-HigA1:SecB^TA^ complex (Fig. 1d). Note that under this condition, the antitoxin only enters into the native gel in the presence of the chaperone. The fact that the antitoxin rapidly aggregates in the absence of chaperone when diluted from urea might explain such a behaviour^12^. In contrast, the *Mtb*-HigA1^W108A/W137A^ C-terminal mutant did not form any detectable complex, even at high antitoxin concentration. Overall, these data show that W108 and W137 in the C-terminal extension of *Mtb*-HigA1 are required for *Mtb*-SecB^TA^ chaperone binding.

To determine whether the C-terminal region of *Mtb*-HigA1 modulates the toxin activation cycle *in vivo*, we first tested the effect of ΔC42 deletion on the *Mtb*-HigA1-dependent inhibition of the *Mtb*-HigB1 toxin, with and without co-expressed *Mtb*-SecB^TA^ chaperone, as described previously^12^. As expected, *Mtb*-HigA1 inhibited the toxin only when the chaperone was co-expressed (Fig. 1e). In sharp contrast, the *Mtb*-HigA1^ΔC42^ mutant can suppress the toxicity of the toxin in the absence of chaperone, albeit less efficiently than *Mtb-*HigA1 WT with co-expressed chaperone at the highest arabinose inducer concentration tested (that is, 0.5%). Note that in both cases toxin expression levels were comparable, indicating that growth rescue is not due to a reduced expression of the toxin (Supplementary Fig. 2). The results obtained with *Mtb*-HigA1^W108A/W137A^ mutant were comparable, although significantly less stringent than the one obtained with the full C-terminal deletion, as judged by the slower growth and the lack of toxin inhibition at intermediate arabinose inducer concentration (that is, 0.1%). In addition, we found that in the presence of *Mtb*-HigA1^W108A/W137A^, but not *Mtb*-HigA1^ΔC42^, a strong overexpression of the chaperone could rescue the bacterial growth defect induced by the toxin (Supplementary Fig. 2). Together, these data indicate that the double tryptophan mutation does not fully mimic the absence of a C-terminal extension. Note that we additionally found that *Mtb*-HigA1^W108A^ single mutant was still assisted by the chaperone, although poorly at high toxin expression level, while *Mtb*-HigA1^W137A^ behaved like *Mtb*-HigA1 WT (Supplementary Fig. 2), indicating that both tryptophan residues could somehow contribute to *Mtb*-SecB^TA^ binding.

To further confirm the role of this region in a mycobacterial host, both *Mtb*-HigA1^ΔC42^ and *Mtb*-HigA1^W108A/W137A^ mutants were compared with *Mtb*-HigA1 WT for toxin inhibition in the closely related non-pathogenic mycobacterium *M. smegmatis*. In this experimental setting, *Mtb*-HigBA1 was present on a multi-copy plasmid under an acetamide-inducible promoter and the chaperone was integrated as a single chromosomal copy and under the control of an anhydrotetracycline-inducible promoter. Under such stringent condition, *M. smegmatis* only tolerates the presence of *Mtb*-HigBA1 when anhydrotetracycline is added to induce chaperone expression (Supplementary Fig. 3). Note that, although *M. smegmatis* growth was rescued by the chaperone when anhydrotetracycline inducer was added, we found that colonies generally grew more slowly when compared with the empty vector, suggesting that chaperone expression level might be limiting in this case. Strikingly, deletion of the C-terminal extension renders the antitoxin fully active and chaperone-independent (compare ΔC42 with WT), while *Mtb*-HigA1^W108A/W137A^ mutant remains inactive, as observed in *E. coli* with 0.1 and 0.5% arabinose (see below for comparison with other mutants).

These *in vivo* results are in agreement with the interaction data described above and show that the C-terminal extension in the antitoxins represents a chaperone addition (ChAD) region within the antitoxin.

### The ChAD region prevents *Mtb*-HigA1 folding

**Figure 2:**
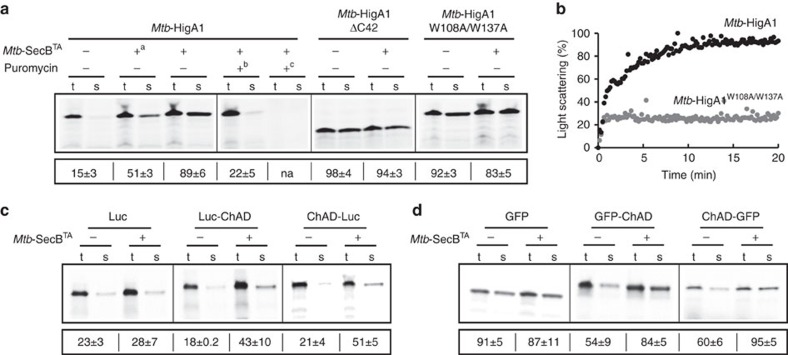
The chaperone facilitates the folding of *Mtb*-HigA1 and other unrelated proteins only in the presence of ChAD. (**a**) Folding of newly translated *Mtb*-HigA1 relies on *Mtb*-SecB^TA^ when the ChAD extension of *Mtb*-HigA1 is present. *Mtb*-HigA1, *Mtb*-HigA1^ΔC42^ and *Mtb*-HigA1^W108A/W137A^ were independently expressed in a cell-free translation system with or without *Mtb*-SecB^TA^. Translation products were labelled with [^35^S]methionine. *Mtb*-SecB^TA^ concentrations were 8 μM otherwise stated (+^a^ indicates 4 μM) and reactions were performed for 1 h at 37 °C. When indicated, 0.4 mM of puromycin was added, either from the start of the translation reaction (+^c^) or 30 min after (+^b^), to release ribosome-bound nascent chains for 1 h, and the chaperone was then added for 1 h at 37 °C. After translation, the total (t) and soluble (s) fractions were separated on SDS–PAGE and quantified by phosphorimager. The numbers below the electrophoretic pattern represent the mean solubility values (%), calculated by the ratio of the amount of translation products in the soluble (s) and total (t) fractions obtained from three different translation experiments. The s.d. is indicated. (**b**) *In vitro* aggregation kinetics of urea-denatured *Mtb*-HigA1 and *Mtb*-HigA1^W108A/W137A^ (4 μM) followed at 25 °C by monitoring light scattering at 355 nm. (**c**) Effect of *Mtb*-SecB^TA^ on the solubility of nascent luciferase (Luc) and chimeric luciferase containing C-terminal ChAD (Luc-ChAD) or N-terminal ChAD-luciferase (ChAD-Luc) and (**d**) on the solubility of nascent GFP and chimeric GFP-C-terminal ChAD (GFP-ChAD) and N-terminal ChAD-GFP (ChAD-GFP), as performed in **a**, with or without *Mtb*-SecB^TA^ (8 μM). Full phosphorimager images for **a**,**c** and **d** are shown in Supplementary Fig. 8.

Our results suggest that the ChAD region of *Mtb*-HigA1 can directly modulate the folding of the antitoxin. In this case, a prior interaction with the SecB-like chaperone becomes a necessary step towards toxin inhibition. To address such hypothesis, we monitored the effect of the chaperone on the folding of newly synthesized WT and mutant antitoxins, using a cell-free coupled transcription/translation *in vitro* assay^17^. The results in Fig. 2a show that newly synthesized *Mtb*-HigA1 mainly aggregates in the absence of chaperone, and that increasing concentrations of chaperone efficiently prevent its aggregation. This is in agreement with the known chaperone function of *Mtb*-SecB^TA^ (ref. 12). In sharp contrast, the *Mtb*-HigA1^ΔC42^ mutant is soluble and its solubility is not modulated by the chaperone. Similar results were obtained for *Mtb*-HigA1^W108A/W137A^. This indicates that the ChAD sequence affects the folding of newly synthesized antitoxin and renders it chaperone-dependent. To confirm such observation, we performed an *in vitro* aggregation assay with purified urea-denatured *Mtb*-HigA1 and *Mtb*-HigA1^W108A/W137A^ and show that in contrast with the rapidly aggregating *Mtb*-HigA1 WT protein, the double tryptophan mutant does not aggregate under these conditions (Fig. 2b). These data support a model in which the ChAD sequence imposes the need for a SecB-like chaperone. Moreover, an early interaction of the chaperone with the nascent antitoxin might be a prerequisite step towards the formation of an active antitoxin, as adding the chaperone once protein synthesis is completed and nascent chains released does not resolve antitoxin aggregates (Fig. 2a).

Next, we asked whether ChAD region exclusively modulates the folding of TAC antitoxins or whether it could somehow affect the proper folding of other proteins. To answer this, the ChAD sequence was grafted to the C-terminal end of two different unrelated model proteins, namely, luciferase (62 kDa) and green fluorescent protein (GFP; 27 kDa), and solubility of these constructs was assessed using the cell-free system described above. We found that ∼23% of luciferase and 91% of GFP were soluble in the absence of grafted ChAD sequence (Fig. 2c,d), which is coherent with the previous work using a similar *in vitro* system with luciferase^18^. In both cases, adding ChAD sequence reduces the fraction of soluble proteins in the absence of chaperone with a significantly more pronounced effect for GFP-ChAD chimeras. Strikingly, the presence of the chaperone significantly increases solubility of both proteins, thus partially recapitulating the effect of ChAD extension on the antitoxin. Furthermore, adding ChAD to the N-terminal end of either luciferase or GFP induces a similar effect, with the solubilization by the chaperone being slightly more efficient in both cases, reaching up to 51% for ChAD luciferase and 95% for ChAD-GFP (Fig. 2c,d). This could suggest that an earlier co-translational interaction of the SecB-like chaperone with N-terminal exposed ChAD sequence^19^ might improve aggregation prevention. Alternatively, this could also solely be due to the steric effects induced by the ability of the chaperone to bind.

Together these results show that the ChAD extension indeed imposes the need for a dedicated SecB chaperone. Yet, the stabilizing/destabilizing effect of ChAD with or without chaperone appears to be significantly more pronounced for *Mtb*-HigA1 than for the two unrelated model proteins used herein, suggesting some specificity for the antitoxin.

### Chaperone-binding site localizes in the first repeat of ChAD

**Figure 3:**
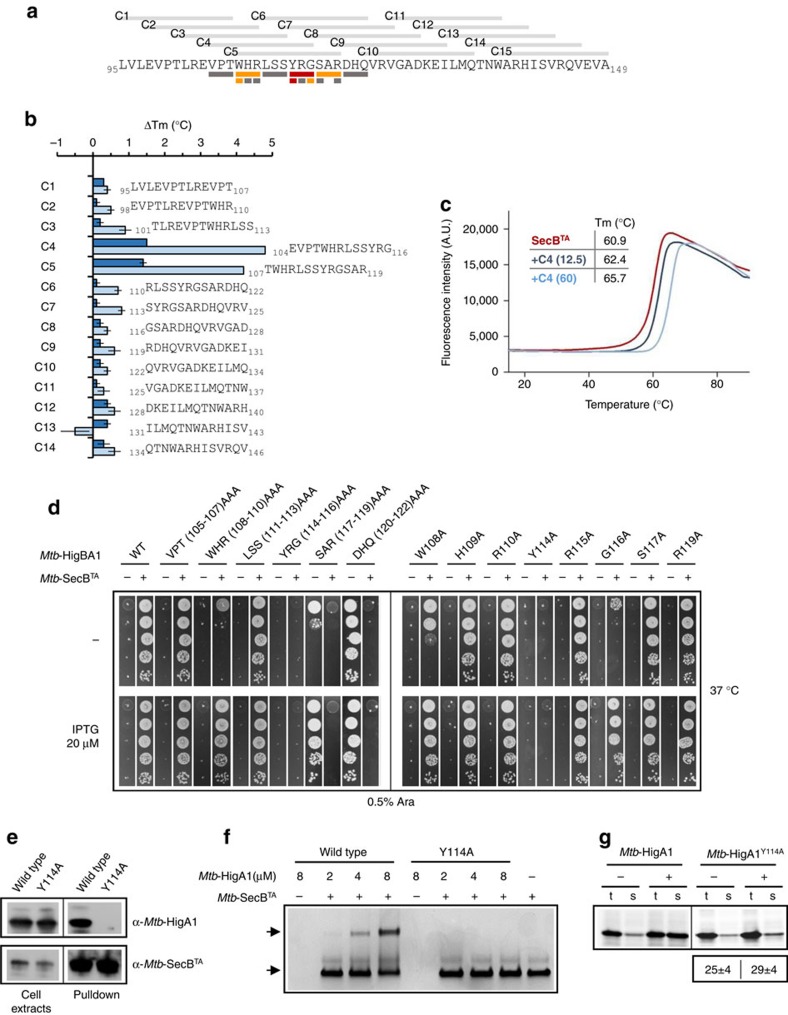
Primary *Mtb*-SecB^TA^ chaperone-binding site of ChAD. (**a**) Thirteen-mer peptides (C1–C15) covering the entire ChAD sequence are depicted on top of the amino-acid sequence. The rectangles displayed under specific regions of the ChAD sequence represents the amino acids (single or triple) that have been changed for alanine in the experiment described in **d**. The grey rectangles represent mutations without apparent phenotype, the orange rectangles represent mutations with partial inactivation and the red rectangles represent mutations with the most severe phenotype, see **d** below. (**b**) DSF analysis of *Mtb*-HigA1 peptide binding to *Mtb*-SecB^TA^. The thermal stability of *Mtb*-SecB^TA^ was monitored in the presence of each peptide. *T*_m_ values were deduced from the fluorescence curves recorded using a temperature gradient from 15 to 90 °C. Shifts in melting temperature are shown at molar ratios of 12.5:1.0 (dark blue bars) and 60:1.0 (light blue bars) of peptides:*Mtb*-SecB^TA^ chaperone. The means and s.e.m.'s of three replicates are shown. (**c**) Representative DSF curves (fluorescence versus temperature gradient) for a temperature gradient from 15 to 90 °C for *Mtb*-SecB^TA^ alone (red) or in the presence of C4 peptide at molar ratios of 12.5:1.0 (dark blue curve) and 60:1.0 (light blue curve) of peptides: *Mtb*-SecB^TA^. (**d**) Suppression of *Mtb*-HigB1 toxicity by *Mtb*-HigA1 and its triple and single alanine mutant derivatives with or without *Mtb*-SecB^TA^ chaperone. Plates were incubated at 37 °C overnight. (**e**) *In vivo* pulldown of His-tagged *Mtb*-SecB^TA^ and *Mtb*-HigA1 (wild type) or *Mtb*-HigA1^Y114A^ (Y114) proteins was revealed using anti-*Mtb*-HigA1 or anti-*Mtb*-SecB^TA^ antibodies. Full blots for **e** are shown in Supplementary Fig. 6. (**f**) *In vitro* native PAGE separation of complexes between *Mtb*-HigA1 or *Mtb*-HigA1^Y114A^, at 2, 4 and 8 μM and *Mtb*-SecB^TA^ (16 μM), as performed in Fig. 1. Full gel for **f** is shown in Supplementary Fig. 7. (**g**) *Mtb*-HigA1^Y114A^ expressed in a cell-free translation system is not solubilized by *Mtb*-SecB^TA^. Experiments were performed as described in Fig. 2a with or without chaperone (8 μM). Full phosphorimager images for **g** are shown in Supplementary Fig. 8.

Next, an *in vitro* approach was used to identify the region of *Mtb-*ChAD that is important for interaction with its dedicated *Mtb*-SecB^TA^ chaperone. Fifteen 13-mer peptides, each overlapping by three residues and encompassing the whole *Mtb*-HigA1 ChAD region (Fig. 3a), were tested for their ability to stabilize the chaperone, using differential scanning fluorimetry (DSF). Strikingly, addition of either C4 or C5 peptides to *Mtb*-SecB^TA^ led to a significant thermal shift (Δ*T*_m_) in a dose-dependent manner (Fig. 3b). At a molar ratio of 12.5:1.0 (peptide:chaperone), we found that Δ*T*_m_ values were +1.5 °C for C4 and +1.4 °C for C5, while at a molar ratio of 60:1.0 (peptide:chaperone), values reached +4.8 and +4.2 °C for C4 and C5, respectively. The strong positive shifts indicate that the C4 and C5 peptides bind the chaperone, likely stabilizing it. Representative melting temperature curves obtained for *Mtb*-SecB^TA^ in the presence of C4 are shown in Fig. 3c. In comparison, thermal shifts for other peptides at the highest molar ratio did not exceed +0.9 °C. Note that peptide C15 could not be solubilized under the conditions used in this work and was thus discarded from this analysis. Together, these results indicate that C4 and C5 peptides likely contain the main chaperone-binding determinants. Noticeably, the 10 amino acids common to both peptides, that is, _107_TWHRLSSYRG_116_ contain the first of the two similar amino-acid stretches present in *Mtb*-ChAD that resemble *E. coli* SecB-binding motifs isolated by peptide-binding scan (see above)^16^.

To further delineate the interacting amino acids, we first performed a triple alanine scanning of the residues covering these two peptides and tested their chaperone dependence *in vivo* in *E. coli* (Fig. 3d). Among these mutants, we found that both WHR(108–110)AAA and SAR(117–119)AAA are partly deficient in suppressing growth inhibition by the toxin, somehow similar to what was observed for the single W108A mutant (Fig. 3d; Supplementary Fig. 2). The YRG(114–116)AAA shows the most severe phenotype, that is, a complete lack of suppression, even at the highest chaperone induction level (Fig. 3d; left panel). We next performed a single alanine scanning of the residues in each triplet of amino acids that had a defect in toxin inhibition and found that only W108A, Y114A and G116A mutants were defective at high toxin expression levels and at the lowest chaperone induction level. Strikingly, only Y114A shows a severe effect that is strictly independent of the chaperone at any inducer concentrations tested, thus highlighting Y114 as a key residue in the ChAD/chaperone complex formation. Accordingly, both *in vivo* pulldown and in *in vitro* native gel separation assays performed with *Mtb*-HigA1^Y114A^ revealed that the mutation indeed abolishes the interaction with the chaperone (Fig. 3e,f). In agreement with such findings, we found that *Mtb*-HigA1^Y114A^ did not inhibit the toxin in *M. smegmatis*, even in the presence of the chaperone (Supplementary Fig. 3). In contrast with the full deletion of the C-terminal ChAD region (that is, *Mtb*-HigA1^ΔC42^ mutant), *Mtb*-HigA1^Y114A^ did not inhibit the toxin under all the conditions tested, thus suggesting that the mutation might affect chaperone binding but not the solubility of the protein, as observed for *Mtb*-HigA1^ΔC42^ and *Mtb*-HigA1^W108A/W137A^ (Fig. 2a). Accordingly, *Mtb*-HigA1^Y114A^ synthesized in the cell-free transcription/translation *in vitro* assay was not efficiently solubilized by the mutation or by addition of the chaperone (Fig. 3g). Further *in vitro* aggregation assay confirmed that the Y114A mutation does not significantly affect aggregation of the antitoxin (Supplementary Fig. 4).

Together, the results presented here suggest that chaperone-binding principally occurs within the first _108_WHRLSSYRG_116_ stretch of amino acids of ChAD, with several amino acids (W108; Y114 and G116) showing various defects *in vivo* when mutated for alanine. The fact that the double W108A/W137A mutation initially designed before the peptide screen has a more severe phenotype than the single W108 and W137 mutants (Supplementary Fig. 2), or even than the WHR(108–110)AAA triple mutant (Supplementary Fig. 3 in *M. smegmatis*; Fig. 3d), suggests that, although not detected by DSF as the main chaperone-binding region, the second motif might also contribute to the recruitment of the chaperone *in vivo*, either as an auxiliary binding site or by further destabilizing the antitoxin. More work is warranted to identify the precise mechanism by which an acquired *Mtb*-ChAD sequence can modulate the folding of the antitoxin that carries it.

### Chaperone addiction among TAC systems shows specificity

As stated above, ChAD sequences are variable both in length and sequence (Supplementary Fig. 1). This suggests that some specificity between a ChAD sequence and its associated SecB-like chaperone might exist. To perform such partner specificity analysis, four different TAC systems were used: the *Mtb-*TAC (*Mtb*-HigBA1/SecB^TA^) and three previously uncharacterized putative TAC systems, namely, the *Mmet-*TAC from *Methylomonas methanica* strain MC09 with a TA couple belonging to the HicAB family, the *Vcho*-TAC from *Vibrio cholerae* strain RC385 with a TA couple belonging to the MqsRA family and the *Glov*-TAC from *Geobacter lovleyi* strain ATCC BAA-1151 with a TA couple belonging to the HigBA family (Supplementary Fig. 5). These new TAC systems are thus representatives of the three different antitoxin families found associated with a SecB-like chaperone in bacterial genomes^10^,^11^.

The putative *Mmet-*, *Vcho-* and *Glov*-TAC systems were cloned under the same expression system used for *Mtb-*TAC^12^ and tested for their ability to function as TA controlled by a SecB-like chaperone, using our *E. coli* growth inhibition/rescue assay. The results presented in Supplementary Fig. 5 show that the three new tripartite systems indeed behave as bona fide TAC, that is, inhibition of the toxin by the antitoxin requires the chaperone. Importantly, these results demonstrate that association of a SecB-like chaperone with TA systems is functionally conserved, and that the dedicated chaperones can indeed assist antitoxins belonging to distinct antitoxin families. Furthermore, as observed for *Mtb*-SecB^TA^, overexpression of the SecB-like chaperones from *M. methanica*, *V. cholerae* or *G. lovleyi* can replace the *E. coli* solitary SecB, as judged by the suppression of the cold-sensitive phenotype of the *E. coli* Δ*secB* mutant (Supplementary Fig. 5). This suggests that these chaperones could work in protein export as well. Steady-state expression levels of the SecB-like chaperones in the presence of 50 μM of isopropyl-β-D-thiogalactoside (IPTG) inducer are shown in Supplementary Fig. 5.

**Figure 4:**
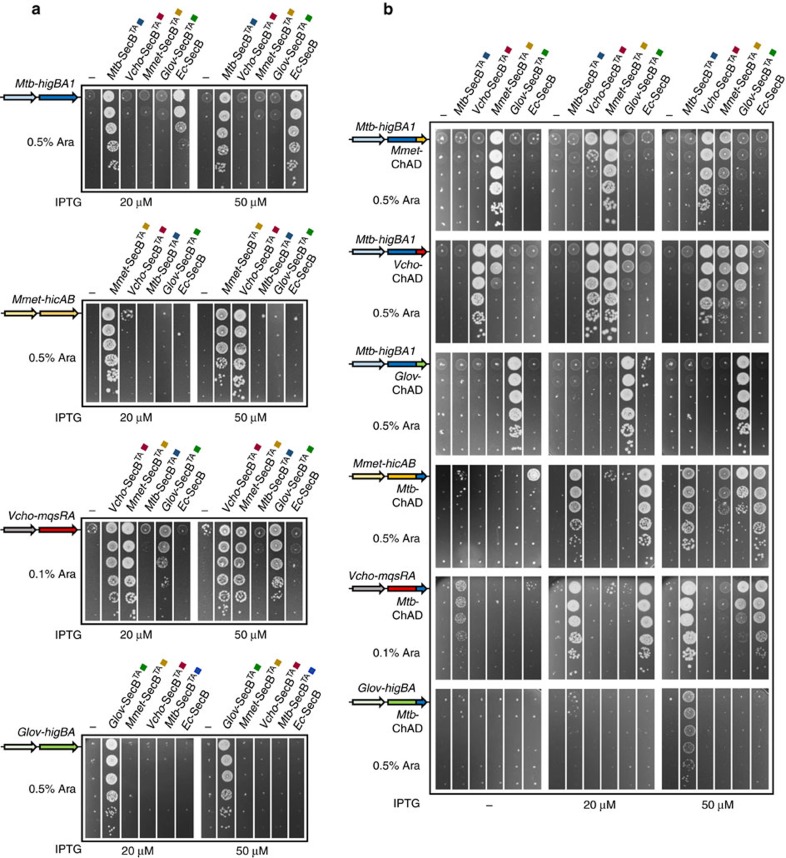
SecB/ChAD addiction modules of TAC systems are specific. (**a**) *Mtb*-SecB^TA^, *VchoA*-SecB^TA^, *Mmet*-SecB^TA^ and *Glov*-SecB^TA^ chaperone specificity towards their cognate TA pair. Strain W3110 Δ*secB* was co-transformed with plasmid pK6-*Mtb-*HigBA1, pK6-*VchoA*-MqsRA, pK6-*Mmet*-HicAB or pK6-*Glov*-HigBA and pSE-based plasmids harbouring different SecB-like chaperones as indicated. Double transformants were grown to mid-log phase, serially diluted and spotted on LB–ampicillin–kanamycin agar plates containing arabinose and IPTG inducers as indicated. Plates were incubated at 37 °C overnight. (**b**) Functional inter-species transfer of SecB/ChAD-specific pairs within TAC systems using antitoxin chimeras. Strain W3110 Δ*secB* was co-transformed with pK6-based plasmids containing chimeric TA systems in which the putative ChAD regions have been swapped, and pSE derivatives harbouring different SecB-like chaperones as indicated, and analysed by spot tests as in **a**.

The four functional TAC systems described here were used to investigate partner specificity by co-expressing TA pairs with non-cognate chaperones. Remarkably, none of the non-cognate chaperones tested can control *Mtb*-HigBA1, even at high IPTG concentration (Fig. 4a, upper panel). Reciprocally, overexpression of *Mtb-*SecB^TA^ is not sufficient to rescue bacterial growth in the presence of *Mmet*-HicAB, *Vcho-*MqsRA or *Glov*-HigBA, thus revealing a robust specificity of *Mtb-*SecB^TA^ for its cognate antitoxin. In contrast, substantial overlap was observed with both *Mmet*- and *Vcho*-SecB^TA^ chaperones, as judged by their ability to control non-cognate *Vcho-*MqsRA and *Mmet*-HicAB, respectively (Fig. 4a, second and third panels). Nevertheless, control of *Mmet*-HicAB by the non-cognate *Vcho*-SecB^TA^ was not as efficient as the one obtained with the native chaperone and thus required higher induction of chaperone. The fact that these two chaperones share high sequence identity at the amino-acid level, that is, ∼53% (compared with 16%, 17% and 19% for *Vcho-*SecB^TA^/*Mtb-*SecB^TA^, *Mmet-*SecB^TA^/*Mtb-*SecB^TA^ and *Glov-*SecB^TA^/*Mtb-*SecB^TA^, respectively), is in line with such functional overlap. Yet, overexpression of *Glov*-SecB^TA^ controlled the non-cognate *Vcho-*MqsRA (Fig. 4a, third panel) but not *Mmet*-HicAB or *Mtb*-HigBA1, in spite of the low sequence identity between the two chaperones (14%). In contrast, none of the non-cognate TAC chaperones could control *Glov*-HigBA (Fig. 4a, bottom panel). Together, these results highlight significant degree of specificity between the chaperones and their cognate TA system.

Next, we asked whether TAC SecB-like chaperones could be redirected to a different TAC, by swapping the ChAD sequence from one TAC to another. To this end, the ChAD sequence of *Mtb*-HigA1 was replaced by the putative C-terminal sequence of *Mmet*-HicB, *Vcho*-MqsA or *Glov-*HigA (corresponding to amino acids 119–159, 136–176 and 86–115, respectively; Supplementary Fig. 5) and the resulting chimeras were tested for specific chaperone partnership in our *in vivo* toxin inhibition assay. Note that the ChAD regions of the TAC antitoxins display no significant similarity (Supplementary Fig. 5). Remarkably, the results presented in Fig. 4b show that grafting *Mmet*-, *Vcho*- or *Glov*-ChAD sequence to *Mtb*-HigA1 results in a functional transfer of *Mmet*, *Vcho* or *Glov* chaperones to *Mtb*-HigBA1. In addition, similar cross-talks as in Fig. 4a were observed between a grafted ChAD sequences and non-cognate chaperones. To further confirm the functional transfer of chaperone addiction, we performed the reverse experiment in which the *Mtb-*ChAD sequence replaces the C-terminal extensions of *Mmet*, *Vcho* or *Glov* antitoxins. In agreement with the proposed specificity of ChAD/SecB^TA^ modules, the results show that the three chimeric TA systems have lost their dependence on their original chaperones and now rely on the *Mtb*-SecB^TA^ chaperone (Fig. 4b). Note that in the case of the *Glov*-HigA-*Mtb*-ChAD chimera, a higher concentration of IPTG inducer was needed to inhibit the growth defect (Supplementary Fig. 5). In addition, at the highest IPTG inducer concentration used, we found that *Mmet*-SecB^TA^ and *Glov*-SecB^TA^ chaperones partially controlled the chimeric *Mmet-*HicAB with grafted *Mtb*-ChAD, thus suggesting some weak binding to the chimeric antitoxin under such conditions. Although such chaperone expression levels are largely beyond the level required for a cognate chaperone to inhibit toxicity^12^, these results might still represent a partially convergent mechanism of ChAD/chaperone interaction between these pairs. In parallel, we also examined whether the solitary export chaperone SecB of *E. coli* exhibits some specificity towards certain ChAD sequences as well. SecB was co-expressed with each TA and TA chimera described above and tested in our *in vivo* toxin inhibition assay. The results from Fig. 4a,b clearly show that at high expression level, SecB efficiently controls *Mtb*-HigBA1, as shown previously^12^, and the two *Mmet* and *Vcho* chimeras containing the ChAD extension of *Mtb*-HigA1, but has no effect on all the other *Mmet*, *Vcho* and *Glov* constructs. This suggests that SecB specifically recognizes and binds the ChAD sequence of *Mtb*-TAC (Fig. 1a).

Together, these data indicate that TAC chaperones are not necessarily interchangeable *in vivo*, and that a specific ChAD/SecB^TA^ module can be transferred as an autonomous entity to control the activation cycle of bacterial toxins. Importantly, it also shows that certain SecB^TA^ chaperones have the ability to efficiently assist antitoxins that belong to very distant families.

### Chaperone addiction is transferable

**Figure 5:**
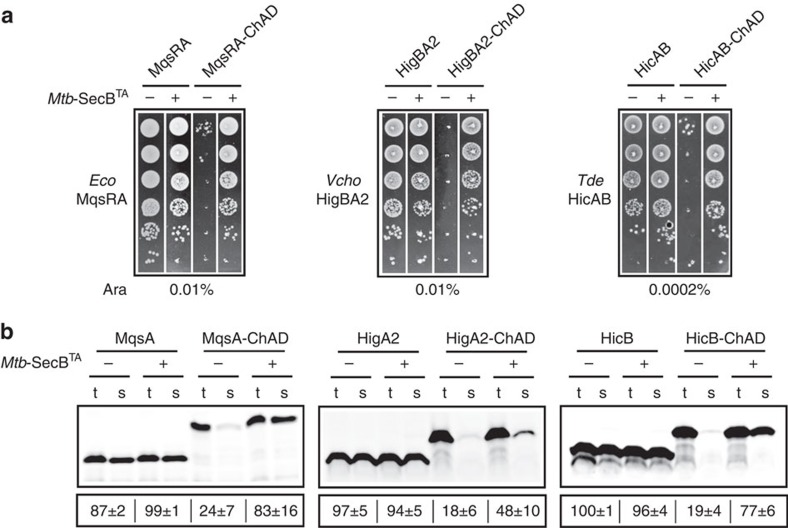
Chaperone addiction module can be transferred to classic two-component TA systems. (**a**) *In vivo Mtb*-SecB^TA^ addiction of classical two-component TA systems belonging to three different TA families in which antitoxins have been fused to the *Mtb*-ChAD. Strain DLT1900 was co-transformed with plasmid pSE vector (−) or pSE-*Mtb*-SecB^TA^ (+) and pK6-*Eco*-MqsRA, pK6-*Eco*-MqsRA-ChAD, pK6-*Vcho*-HigBA2, pK6-*Vcho*-HigBA2-ChAD, pK6-*Tde*-HicAB or pK6-*Tde*-HicAB-ChAD. Double transformants were grown to mid-log phase, serially diluted and spotted on LB–ampicillin–kanamycin agar plates with arabinose inducer as indicated. Plates were incubated at 37 °C overnight. (**b**) Effects of *Mtb*-SecB^TA^ on the solubility of *Eco*-MqsA, *Vcho*-HigA2, *Tde*-HicB wild type or chimeras containing the *Mtb*-ChAD region, namely, *Eco*-MqsA-*Mtb*-ChAD (MqsA-ChAD), *Vcho*-HigA2-*Mtb*-ChAD (HigA2-ChAD) and *Tde*-HicB-*Mtb*-ChAD (HicB-ChAD), synthesized using a reconstituted cell-free translation system with or without *Mtb*-SecB^TA^ chaperone (8 μM) as performed in Fig. 2a. Full phosphorimager images for **b** are shown in Supplementary Fig. 8.

Since chaperone addiction is transferable among TAC systems, we asked whether classical two-component systems from the HigBA, MqsRA or HicAB families could become addicted to chaperone as well. We chose one representative of each type. Two of them were previously characterized, namely, HigBA2 of *V. cholerae*^20^ and MqsRA of *E. coli*^21^, whereas the third one that belongs to the HicAB^22^ TA family, the TDE0482-0481 locus of *Treponema denticola*, has not been studied yet. We cloned them with or without grafted ChAD sequence of *Mtb*-HigA1, into the pMPMK6 plasmid under the control of arabinose inducer, as performed with *Mtb*-HigBA1 from TAC. All constructs were tested in our *in vivo* toxin inhibition assay (Fig. 5a). As expected, expression of each of the three two-component TA systems does not affect bacterial growth. This is in agreement with a bona fide two-component TA, in which the toxin is efficiently inhibited by its co-transcribed cognate antitoxin. Strikingly, grafting the ChAD sequence to these TA systems completely inhibits antitoxin function and co-expression of the *Mtb*-SecB^TA^ chaperone fully restores it. This demonstrates that chaperone addiction can indeed be transferred to classical two-component TA systems *in vivo*. Next, we investigated the effect of the chaperone on the folding of newly synthesized chimeric antitoxins *in vitro*, as performed in Fig. 2. Remarkably, grafting the ChAD sequence to these chaperone-independent antitoxins severely affects their folding in the absence of the chaperone, in a manner comparable to that of *Mtb*-HigA1 (Fig. 5b). Reciprocally, such antitoxin chimeras were significantly solubilized by adding purified *Mtb*-SecB^TA^ chaperone. Taken together, these results demonstrate that chaperone addiction phenomenon can be transferred to a variety of chaperone-independent two-component TA systems.

## Discussion

This work reveals the underlying mechanism of chaperone addiction of conserved TA pairs. It shows that short C-terminal amino-acid extensions within TAC antitoxins (herein named ChAD) are specifically recognized by a dedicated SecB chaperone, which facilitates antitoxin folding and its subsequent interaction with the toxin. Such a mechanism involving SecB/ChAD-specific recognition likely confers an additional level of post-translational regulation of TA systems, in which titrating out the chaperone could trigger toxin activation. The fact that TAC chaperones can replace the *E. coli* export chaperone SecB suggests that stress-induced protein aggregation or *de novo* synthesis of specific secretory proteins could hijack the chaperone and ultimately lead to the activation of the toxin for adaptive purposes^12^. When bound to the antitoxin, the chaperone could also act as a transcriptional co-repressor of *TAC*, similarly to what was previously described for the DnaK and GroEL chaperones, known to act as co-repressors of heat-shock genes by modulating the activity of the transcriptional repressors HspR and HrcA, respectively^23^,^24^,^25^. Such a mechanism would ensure a robust repression of toxin synthesis and directly link toxin expression to specific changes that do not only rely on proteases activation^26^,^27^,^28^,^29^ but on chaperone availability as well^12^. Whether a SecB-like chaperone directly modulates accessibility of its cognate antitoxin to proteases under certain circumstances and whether the ChAD sequence is involved in this process remains to be determined.

This work also demonstrates that ChAD/SecB pairs can function as independent modules that can be grafted to classical two-component TA systems and render them chaperone-dependent. Similarly, such SecB chaperone addiction phenomenon is also transferable to unrelated proteins and in this case, the ChAD sequence can also be functionally grafted at the N-terminal part of these proteins. Grafting chaperone/ChAD modules to specific protein targets might thus represent a valuable tool to optimize the expression and folding of difficult heterologous proteins expressed in *E. coli* for biotechnological or medical purposes. Since a significant number of SecB sequences (over 7%) are associated with TA systems, it is very likely that this represents an important reservoir of unexplored specific chaperone/ChAD cognates^11^.

Short signal peptide segments are widely used in many biological processes to target proteins to specific secretory or degradation pathways, and in many cases such pathways are driven by molecular chaperones^30^,^31^,^32^,^33^,^34^,^35^,^36^. More specifically, the N-terminal signal peptides of protein substrates of the Twin–arginine translocation (Tat) pathway possess an hydrophobic region that is generally recognized by specific chaperones, either REMPs (redox enzyme maturation proteins) or trigger factor derivatives, known to maintain Tat substrates in a conformation compatible for further maturation and folding, to prevent their degradation, and to facilitate their subsequent targeting to the translocase^37^,^38^,^39^,^40^. Although Tat signal peptides appeared to be primary chaperone-binding sites, it has been shown that the chaperones can also interact with the mature part of their specific Tat substrate, which could be the case for SecB^TA^ chaperones and TAC antitoxins as well^41^. Whether Tat signal peptide stimulates aggregation of Tat substrates and whether such aggregation is inhibited by dedicated REMPs is not known. In the case of the maltose-binding protein precursor (pre-MBP), a known SecB-dependent substrate in *E. coli*, it has been shown that the presence of the N-terminal signal peptide kinetically interfere with the folding to mature species, and that pre-MBPs were more prone to aggregation than mature MBPs, thus somehow mirroring the effect of ChAD on the folding of antitoxins, GFP and luciferase^42^,^43^. Yet, despite the fact that pre-MBP is a *bona fide E. coli* SecB substrate, its signal peptide is not the primary chaperone-binding site, as it seems to be the case for ChAD/SecB^TA^.

Remarkably, all TAC chaperones tested so far efficiently complement for the cold-sensitive phenotype of the *E. coli secB* mutant, although they show significant specificity towards their respective ChAD sequence^10^,^12^ (this work). This suggests that SecB chaperones can efficiently be rerouted to accommodate short C-terminal ChAD extensions without losing their ability to functionally bind a wide range of presecretory substrates. The fact that SecB possesses a remarkably large substrate-binding surface that can accommodate very long fragments within polypeptide substrates (up to 150 amino acids in length) indeed suggests that discreet changes within this surface could specialized the chaperone towards the ChAD sequence of an antitoxin without detectably modifying its overall generic chaperone properties^41^,^44^. More work is warranted to identify such binding area on SecB^TA^ chaperones.

## Methods

### Bacterial strains and culture conditions

*E. coli* strains BL21(DE3) (Novagen), W3110, W3110 *secB*::Cm^R^ (ref. 45) and DLT1900 (ref. 46), and *M. smegmatis* MC^2^155 (strain ATCC 700084) have been described. Protease-deficient derivative of W3110 was constructed as follows. The Δ*clpQ*::KanR allele from strain JWK3903 (Keio collection) was first moved in W3110 using P1-mediated transduction and by subsequent removing of the kanamycin resistance cassette using plasmid pCP20, as described^47^. The Δ(*clpPX-lon*)1196::Cm^R^ allele from KY2266 (ref. 48) was then moved into W3110 Δ*clpQ*, leading to strains GP1119. Bacteria were routinely grown in LB medium supplemented when necessary with kanamycin (50 μg ml^−1^) or ampicillin (50 μg ml^−1^).

### Plasmid constructs

Plasmids pETDuet-1 (Novagen), pSE380, pSE380ΔNcoI^49^, pSE-SecB^50^, pSE-*Mtb*-SecB^TA^ (ref. 12), pMPMK6 (ref. 51) and pK6-*Mtb*-HigBA1 (ref. 12) have been described. To construct plasmid pSE-_6HIS_*Mtb*-SecB^TA^, the NcoI/BamHI-digested fragment containing the N-terminal HIS_6_-tagged *Mtb*-secB^TA^ from pET15b-*Mtb*-secB^TA^ (ref. 12) was subcloned into NcoI/BamHI-digested pSE380.

Plasmid derivatives encoding *Mmet*, *Vcho* and *Glov*-TAC chaperones were constructed as follows. For plasmid pSE-*Mmet*-SecB^TA^, the 486-nt-long *Metme_2811* gene was PCR-amplified from *M. methanica* MC09 genomic DNA (a gift of Andrew Crombie, University of East Anglia, Norwich, UK) using primers CMmetFORE/N: 5′-ttgaattccatatgaacgcaaatctacaaaaagcc-3′ and CMmetREVH/Bam: 5′-ttaagcttggatcctaatccttctcggcatttgtag-3′, and cloned as an EcoRI–HindIII fragment into pSE380ΔNcoI digested with the same enzymes. For plasmid pSE-*Vcho*-SecB^TA^, the 462-nt-long *VCRC385_03680* gene was PCR-amplified from *V. cholerae* RC385 genomic DNA (a gift of Rita Colwell, University of Maryland, College Park, USA) using primers CVchoAFORE/N: 5′-ttgaattccatatgtctaaaaaagtatcaaaatc-3′ and CVchoAREVH/Bam: 5′-ttaagcttggatccttactgtttgctaaattgcacag-3′. PCR fragment was digested with EcoRI–HindIII and ligated into pSE380ΔNcoI digested with the same enzymes. For plasmid pSE-*Glov*-SecB^TA^, the 432-nt-long *Glov_3490* gene was PCR-amplified from *Geobacter lovleyi* DSM 17,278 genomic DNA (DSMZ, Braunschweig, Germany) using primers CGlovFORE/N: 5′-ttgaattccatatatgcccaaggttaaacaaacgcc-3′ and CGlovREVH/Bam: 5′-ttaagcttggatccttattttccggaatggttttgtttg-3′. PCR fragment was digested with EcoRI–HindIII and ligated into pSE380ΔNcoI digested with the same enzymes.

The various luciferase and GFP constructs in pMPMK6-based vector were constructed as follows. For pK6-Luc, the luciferase gene from *P. pyralis* was PCR-amplified using primers LucERINdeI-For: 5′-gagaattccatatggaagacgccaaaaacataaag-3′ and LucBglII-Rev: 5′-gaagatctttacacggcgatctttccgcccttc-3′ and cloned as an EcoRI–BglII fragment into EcoRI–BglII-digested pMPMK6. For plasmid pK6-Luc-*Mtb*-ChAD, encoding the chimeric luciferase protein with the *Mtb*-ChAD C-terminal domain, the *P. pyralis* luciferase gene with no stop codon was PCR-amplified with flanking EcoRI and XbaI sites using primers LucERINdeI-For and LucXbaI-Rev: 5′-gatctagacacggcgatctttccgcccttc-3′, and cloned as an EcoRI–XbaI fragment into pK6-*Mtb*-ChAD fragment digested with the same enzymes. Note that the pK6-*Mtb*-ChAD fragment with flanking EcoRI–XbaI was obtained after PCR amplification with primers 56C1XbaI-For: 5′-gatctagagaagtgcctacgtggcatc-3′ and pMPMK6-EcoRI-Rev: 5′-gtgaattcctcctttcactccatc-3′. To construct pK6-*Mtb*-ChAD-Luc, encoding a chimeric luciferase protein with the grafted N-terminal ChAD domain of *Mtb*-HigA1, the *P. pyralis* luciferase gene and the 3′-region (141 bp) of *Mtb*-*higA1* flanked by EcoRI and HindIII sites were obtained by fusion PCR with an overlapping region using primers C1N-terFORE: 5′-ttgaattcatggaagtgcctacgtggcatc-3′, C1-LucForoverlap: 5′-ggttcggcaggttgaggtggcatctagaatggaa-3′, LucRevREVH: 5′-ttacacggcgatctttccgcccttc-3′ and C1-lucRevoverlap: 5′-gtttttggcgtcttccattctagatgccacctcaacctgccgaacc-3′. The EcoRI–HindIII-digested fragment was then cloned into pK6 digested with the same enzymes.

For plasmid pK6-GFP, the *gfpUV4* gene was PCR-amplified using primers GFPUV4ERINdeI-For: 5′-gagaattccatatgagtaaaggagaagaacttttc-3′ and GFPUVBglII-Rev: 5′-gaagatctttatttgtagagctcatccatgc-3′, and cloned into pK6 digested with EcoRI and BglII. For pK6-GFP-*Mtb*-ChAD, encoding the chimeric GFP with the *Mtb*-ChAD C-terminal domain, the *gfpUV4* gene with no stop codon was PCR-amplified with flanking EcoRI and XbaI sites using primers GFPUV4ERINdeI-For and GFPUV4XbaI-Rev: 5′-gatctagatttgtagagctcatccatgc-3′, and was ligated into EcoRI–XbaI-digested pK6-*Mtb*-ChAD fragment, as described above for pK6-Luc-*Mtb*-ChAD. To construct pK6-*Mtb*-ChAD-GFP, encoding a chimeric GFP with the grafted N-terminal ChAD domain, the *gfpUV4* gene and the 3′ region (141 bp) of *Mtb-higA1* were obtained by fusion PCR with an overlapping region using primers C1N-terFORE, gfpRevREVH: 5′-ttatttgtagagctcatccatgcc-3′, C1-gfpForoverlap: 5′-ggttcggcaggttgaggtggcatctagaatgagtaaaggagaagaac-3′ and C1-gfpRevoverlap: 5′-gttcttctcctttactcattctagatgccacctcaacctgccgaacc-3′.

The plasmids used for the chaperone addiction of classical two-component TA systems were constructed as follows. To construct plasmid pK6-*Vcho*-HigBA2, the 634-nt-long *Vcho-higBA2* operon was PCR-amplified from genomic DNA (a gift from Didier Mazel, Institut Pasteur, Paris, France) with primers HigBA2ERINdeI-For: 5′-gagaattccatatgaaaagtgtatttgtcgaatc-3′ and VC2HigBAREv: 5′-gaagatctttatagctcggctatgtgtg-3′, and was cloned into pK6 using EcoRI and HindIII. For plasmid pK6-*Vcho*-HigBA2-*Mtb*-ChAD, encoding *Vcho*-HigBA2 with the chimeric antitoxin containing the grafted *Mtb*-ChAD region at the C-terminal, the *Vcho*-higBA2 operon with no stop codon was PCR-amplified with flanking EcoRI and XbaI sites using primers HigBA2ERINdeI-For and HigBA2XbaI-Rev: 5′-gatctagatagctcggctatgtgtgacaac-3′, and was cloned as an EcoRI–XbaI fragment into the pK6-*Mtb*-ChAD fragment that was obtained following PCR with primers 56C0XbaFor: 5′-gatctagagaagttcccacgcttcgcgaag-3′ and pMPMK6-EcoRI-Rev, and then was digested with the same enzymes, as described above for pK6-Luc-*Mtb*-ChAD.

Plasmid pK6-*Eco*-MqsRA was obtained following PCR amplification of the 693-nt-long *mqsRA* operon from *E. coli* genomic DNA, using mqsRERI: 5′-gagaatccatggaaaaacgcacaccacatac-3′ and mqsARev: 5′-gaaagcttggatccttaacggatttcattcaatagttc-3′, and cloning into pK6 using EcoRI and HindIII. To construct plasmid pK6-*Eco*-MqsRA-*Mtb*-ChAD, encoding Eco-MqsRA with the chimeric antitoxin containing the grafted C-terminal *Mtb*-ChAD, the *mqsRA* operon was PCR-amplified with flanking EcoRI and XbaI sites using primers mqsRERI and mqsRAXba: 5′-gatctagaacggatttcattcaatagttctg-3′, and was cloned as an EcoRI–XbaI fragment into pK6-*Mtb*-ChAD as described above for pK6-*Vcho*-HigBA2-*Mtb*-ChAD.

For pK6-*Tde*-HicAB, the 570-nt-long long genomic region comprising the *hicA* toxin TDE0482 and the *hicB* antitoxin TDE0481 was amplified by PCR using primers TDE0482FORFusion: 5′-tgaaaggaggaattcatggctagtgttgaaaaaataattg-3′ and TDE0481REVFusion: 5′-ggtatcgataagcttttaagaaagtctataaagaacgtattg-3′, and *T. denticola* DSM14222 genomic DNA (DSMZ, Braunschweig, Germany) as a template. The PCR fragment was then cloned by homologous recombination with the In-Fusion PCR cloning system (Clontech). To obtain plasmid pK6-*Tde*-HicAB-*Mtb*-ChAD, encoding the HicAB system with a chimeric antitoxin containing the C-terminal *Mtb*-ChAD domain, pK6-*Tde*-HicAB was linearized by PCR using primers TDE0481 REV: 5′-agaaagtctataaagaacgtattg-3′ and pK6HindFusion, and was recombined with the PCR fragment corresponding to the 3′ (141 bp) ChAD region of *Mtb*-HigA1 that was obtained by PCR using vector pK6-*Mtb*-HigBA1 as a template and primers TDE0481-C1MtbFOR: 5′-caatacgttctttatagactttcttctagagaagtgcctacgtgg-3′ and C1HigAREVFusion, using the In-Fusion PCR cloning system.

Plasmids used in the SecB^TA^:ChAD specificity experiments were constructed as follows. Plasmid pK6-*Mmet*-HicAB was obtained by PCR amplifying the 773-nt-long genomic region comprising the *hicA* toxin Metme_2813 and the *hicB* antitoxin Metme_2812 using primers TMmetFORE/N: 5′-ttgaattccatatgagtgctaaactacgtatcc-3′ and AMmetREVH/Bam: 5′-ttaagcttggatcctcaggcgtattgtttttctaacgc-3′, and *M. methanica* MC09 genomic DNA as a template. The PCR product was digested with EcoRI–HindIII and was ligated into pK6 digested with the same enzymes. To construct pK6-*Mmet*-HicAB-*Mtb*-ChAD, pK6-*Mmet*-HicAB was linearized by PCR using primers TAMmet-RevFusion: 5′-accctgcgcataaccggcgtaa-3′ and pK6 Hind Fusion: 5′-aagcttatcgataccgtcgacctc-3′, and was recombined to the PCR fragment corresponding to the 3′ (141 bp) ChAD region of *Mtb*-*higA1* that was amplified using vector pK6-*Mtb*-HigBA1 as a template and primers C1HigAForFusion(Mmet): 5′-ggttatgcgcagggtgaagtgcctacgtggcatcgg-3′ and C1HigAREVFusion: 5′-ggtatcgataagcttcatgccacctcaacctgccgaac-3′ using the In-Fusion PCR cloning system.

To construct pK6-*Vcho*-MqsRA, the 981-nt-long genomic region comprising the MqsR toxin VCRC385_03678 and the MqsA antitoxin VCRC385_03679 was PCR-amplified using primers TVchoAFORE/N: 5′-ttgaattccatatggtaaataatgcgataaatg-3′ and AVchoAREVH/Bam: 5′-ttaagcttggatccttagacatagcacgcctctaattc-3′, and *V. cholerae* RC385 genomic DNA as a template. The PCR product was digested with EcoRI–HindIII and was ligated into pK6 digested with the same enzymes. To construct pK6-*Vcho*-MqsRA-*Mtb*-ChAD, pK6-*Vcho*-MqsRA was linearized by PCR using primers TAVcho-RevFusion: 5′-atccaacattaacttatctaa-3′ and pK6HindFusion, and was recombined with the PCR fragment corresponding to the 3′ (141 bp) ChAD region of *Mtb*-*higA1* that was amplified with pK6-Mtb-HigBA1 as a template and primers C1HigAForFusion(Vcho): 5′-aagttaatgttggatgaagtgcctacgtggcatcgg-3′ and C1HigAREVFusion, using the In-Fusion PCR cloning system.

Plasmid pK6-*Glov*-HigBA was obtained by PCR amplifying the 767-nt-long genomic region comprising the *higB* toxin Glov_3492 and the *higA* antitoxin Glov_3491 using primers TGlovFORE/N: 5′-ttgaattccatatgttcataaaaagcctgatacc-3′ and AGlovREVH/Bam: 5′-ttaagcttggatccttaaccttgggcatgacgatatac-3′, and *G. lovleyi* DSM17278 genomic DNA as a template. The PCR product was digested with EcoRI–HindIII and was ligated into pK6 digested with the same enzymes. To construct pK6-*Glov*-HigBA-*Mtb*-ChAD, pK6-*Glov*-HigBA was linearized by PCR using primers TAGlov-RevFusion: 5′-tgcaagagttggggtaaacttc-3′ and pK6 Hind Fusion, and was recombined to the PCR fragment corresponding to the 3′ (141 bp) ChAD region of *Mtb*-*higA1* that was amplified using vector pK6-*Mtb*-HigBA1 as a template and primers C1HigAForFusion(Glov): 5′-accccaactcttgcagaagtgcctacgtggcatcgg-3′ and C1HigAREVFusion, using the In-Fusion PCR cloning system.

For plasmid pK6-*Mtb*-HigBA1-*Mmet*-ChAD, encoding *Mtb*-HigBA1 with a chimeric antitoxin in which the *Mtb*-ChAD region has been replaced by the ChAD region of the *M. methanica* MC09 TAC antitoxin, pK6-*Mtb*-HigBA1 was linearized by PCR using primers HigBA-Rev Fusion: 5′-gcgaagcgtgggaacttcgag-3′ and pK6HindFusion, and recombined using the In-Fusion PCR cloning system, with the PCR fragment corresponding to the 3′ (126 bp) ChAD region of *Mmet*-*hicA* that was amplified using pK6-*Mmet*-HicAB as a template and primers C1MmetFORFusion: 5′-gttcccacgcttcgcttagaagctcgaaatagtagtga-3′ and C1MmetREVFusion: 5′-ggtatcgataagcttcaggcgtattgtttttctaac-3′.

To construct pK6-*Mtb*-HigBA1-*Vcho*-ChAD, encoding *Mtb*-HigBA1 with a chimeric antitoxin in which the *Mtb*-ChAD region has been replaced by the ChAD region of the of *V. cholerae* RC385 TAC antitoxin, the 728-nt-long region comprising *Mtb*-higBA1 and the first 309 nt of *Mtb*-*higA1* of *M. tuberculosis* H37Rv, and the 3′ (126 nt) ChAD region of the TAC antitoxin of *V. cholerae* RC385 were PCR-amplified with a small overlapping region in the middle using primers 1955*-for: 5′-gagaattccatatgccgccccctgatccagccgccatg-3′ HigA-C1VchoFOR: 5′-ctcgaagttcccacgcttcgcgcgggtgttgagcgtaatatc-3′, HigA-C1VchoREV: 5′-gatattacgctcaacacccgcgcgaagcgtgggaacttcgag-3′ and AVchoAREVH/Bam to obtain one unique fragment with flanking EcoRI and HindIII sites using a two-step fusion PCR. This PCR fragment was then cloned into EcoRI–HindIII-digested pK6.

For plasmid pK6-*Mtb*-HigBA1-*Glov*-ChAD, encoding *Mtb*-HigBA1 with a chimeric antitoxin in which the *Mtb*-ChAD region has been replaced by the ChAD region of the *G. lovleyi* DSM17278 TAC antitoxin, pK6-*Mtb*-HigBA1 was linearized by PCR using primers HigBA-Rev Fusion and pK6HindFusion, and recombined using the In-Fusion PCR cloning system, with the PCR fragment corresponding to the 3′ (81 bp) ChAD region of the TAC antitoxin of *G. lovleyi* DSM17278 that was amplified using pK6-*Glov*-HigA as template and primers C1GlovFORFusion: 5′-gttcccacgcttcgcgtggagcctgtctctgaggctg-3′ and C1GlovREVFusion: 5′-ggtatcgataagctttaaccttgggcatgacgatatacc-3′.

The pETDuet-based vectors used for the co-purification experiments were constructed as follows. For pETDuet-*Mtb*-HigA1, the *higA* gene was PCR-amplified from pK6-*Mtb*-HigBA1 using primers 1956-FOR: 5′-gagaattccatatgagcattgacttccctttgggtg-3′ and 1956-REV: 5′-gaagatcttcatgccacctcaacctgccgaac-3′, digested with EcoRI–BglII, and ligated into pETDuet-1 digested with the same enzymes (into multi-cloning site 2). To construct pETDuet-_6HIS_*Mtb*-SecB^TA^-HigA1, His-tagged *Rv1957* gene was PCR-amplified from pET15b-_6HIS_*Mtb*-secB^TA^ using primers T7-FOR: 5′-taatacgactcactatag-3′ and T7-REV: 5′-ctagttattgctcagcgg-3′, digested with NcoI–BamHI and ligated into pETDuet-*Mtb*-HigA1 digested with the same enzymes (into multi-cloning site 1). This construct allows the co-expression of *Mtb*-HigA1 and _6HIS_*Mtb*-SecB^TA^. Plasmid pETDuet-HigA1-_6HIS_SecB^TA^ was used as a template to construct plasmid pETDuet-_6HIS_*Mtb*-SecB^TA^-HigA1^W108A/W137A^, pETDuet-_6HIS_*Mtb*-SecB^TA^-HigA1^Y114A^ and pETDuet-_6HIS_*Mtb*-SecB^TA^-HigA1^ΔC42^ by Quickchange mutagenesis Stratagene, with *higA1* codon change tgg>gcc for W108A,tgg>gcc for W137A, tat>gcc for Y114A and tgg>taa for ΔC42.

Plasmid pLAM12 (ref. 52) encoding the HigB1 toxin and the HigA1 antitoxin either WT or WHR(108–110)AAA mutant was constructed using the In-Fusion HD cloning kit. To this purpose, the pLAM12 plasmid was linearized by PCR, *Mtb-higBA1* was amplified from the pK6-HigBA1 plasmid^12^ and *Mtb-higBA1* WHR(108–110)AAA was amplified from the pK6-HigBA1^WHR-AAA^ plasmid. The pLAM-HigBA1 was then used as a template to construct pLAM-*Mtb*-HigBA1^Y114A^, pLAM-*Mtb*-HigBA1^W108A/W137A^ and pLAM-*Mtb*-HigBA1^ΔC42^, using Quickchange mutagenesis. *M. smegmatis* MC^2^155 pGMCS-*Mtb*-SecB^TA^ strain carries the pGMCS-*Mtb*-SecB^TA^ plasmid integrated into the phage L5 attachment site of the chromosome. This plasmid encodes *Mtb*-SecB^TA^, whose expression is under the control of the *PmyctetO* promoter^53^ and was constructed after cloning in vectors pGMCS of the Gateway system^54^ developed by the groups of Sabine Ehrt and Dirk Schnappinger at Weill Cornell Medical College, New York. The constructs obtained by PCR were sequenced verified using appropriate primers.

### *In vivo* TA control by chaperones

Toxin inhibition assays were performed *in vivo* as described^12^. In brief, fresh transformants of *E. coli* strains co-expressing the chaperones and the TA pairs were grown in LB–ampicillin–kanamycin to mid-log phase, serially diluted and spotted on LB–ampicillin–kanamycin agar plates with or without IPTG and arabinose inducers as indicated in the figure legends. Plates were incubated overnight at 37 °C.

### *In vivo* chaperone assays

Complementation of SecB chaperone activity *in vivo* at 16 °C was performed as described^12^. In brief, fresh transformants of W3110 Δ*secB*::CmR containing pSE derivatives harbouring SecB-like chaperones were grown at 37 °C to mid-log phase in LB, serially diluted and spotted on LB ampicillin agar plates in the absence or presence of IPTG inducer. Plates were incubated at 37 °C overnight or for 5 days at 16 °C.

### Western blot analysis

One millilitre aliquots of the cell cultures was collected at 5,000*g*. Whole-cell extracts were prepared by resuspending the pellets in 1 × SDS loading dye (1/4th volume of the initial OD_600_). Proteins were separated by SDS–polyacrylamide gel electrophoresis (SDS–PAGE) and transferred onto polyvinylidene difluoride membranes (Bio-Rad). Membranes were blocked overnight at 4 °C in 5% nonfat dry milk in PBS containing 0.05% Tween 20. Primary antibodies were anti-Rv1957 (1:1,000) and anti-*Mtb*-HigA1 (1:1,000)^12^, and anti-*Mtb*-HigB1 (1:1,000; raised against amino acids 20–34 and 111–125 of *Mtb*-HigB1; Eurogentec) and anti-Luciferase (1:1,000; Promega). Horseradish peroxidase-conjugated rabbit IgG or anti-goat IgG was used as a secondary antibody. Blots were developed by chemiluminescence using Clarity western ECL substrate (Bio-Rad) with a luminescence analyser (LAS4000, Fuji) and were analysed using Multigauge software (Fuji). Full blots from Fig. 1b,c and Fig. 3e are shown in Supplementary Fig. 6.

### *In vivo* pull-down assays

To test the interaction between *Mtb*-SecB^TA^ and *Mtb*-HigA1 mutants, *E. coli* strain BL21 was transformed with pETDuet derivatives containing _His6_*Mtb*-SecB^TA^ and *Mtb*-HigA1 either WT or mutant. Overnight cultures of transformants were diluted to an OD_600_ of 0.05 and grown at 37 °C to OD_600_ of 0.4 in LB. The expression of *Mtb*-SecB^TA^ and *Mtb*-HigA1 variants was then induced for 2 h with 0.5 mM IPTG. For the interaction between *Mtb*-SecB^TA^ and luciferase chimeric proteins, *E. coli* strain W3110 Δ*clpPX-lon*::Cm^R^ Δ*clpQ* was co-transformed with pSE containing *Mtb*-SecB^TA and^ and pMPMK6 derivatives harbouring Luc_ChAD_Mtb_, or Luc_ChAD_Mtb_^W108A/W137A^. Overnight cultures of fresh transformants were diluted to an OD_600_ of 0.05 and were grown at 37 °C to mid-log phase in LB. The expression of *Mtb*-SecB^TA^ and luciferase was then induced for 90 min with 0.2% arabinose and 50 μM IPTG, respectively. Ni-NTA affinity pull-down assays were carried out as reported^12^. In brief, crude cell extracts were incubated for 2–3 h with equilibrated Ni-NTA beads in 50 mM Tris pH 7.5 containing 150 mM NaCl on a rotator at 4 °C. Bound proteins were eluted with 250 mM imidazole, fractionated by SDS–PAGE on 4–20% Mini-Protean TGX gels (Bio-Rad) and subjected to western blot analysis using anti-*Mtb*-HigA1, anti-*Mtb*-SecB^TA^ or anti-Luciferase antibodies.

### Native gel separation of purified protein complexes

*Mtb*-SecB^TA^ was purified following overexpression from pET15b in *E. coli* BL21(λDE3) as described^12^. Purified *Mtb*-HigA1 WT, W108A/W137A and Y114A (in 20 mM Tris, pH 7.5, 150 mM NaCl, 1 mM dithiothreitol (DTT), 20% glycerol, 8 M urea) were obtained following overexpression from pET15b-_His6_HigA, pET15b-_His6_HigA^W108A/W137A^ and pET15b-_His6_HigA^Y114A^ in *E. coli* BL21(λDE3) from the aggregated fractions, as described^12^. Protein complexes were formed by mixing 2, 4 or 8 μM of unfolded *Mtb*-HigA1 WT, W108A/W137A or Y114A and 16 μM of *Mtb*-SecB^TA^ at 37 °C for 15 min in 40 μl reaction buffer (30 mM HEPES pH 7.5, 40 mM KCl, 7 mM MgAc and 50 mM NaCl). Reaction mixtures were separated using native PAGE on 4–20% Mini-PROTEAN TGX precast gels (Bio-Rad) using Tris/Glycine buffer, and run at 125 V for 2 h at room temperature. Gels were stained with InstantBlue stain.

### Differential scanning fluorimetry

DSF was used to characterize the thermal stability of the enzyme in the absence and presence of peptides selected from the *Mtb*-HigA1 sequence. In this case, 13-mer peptides were synthesized by Schafer-N (Copenhagen, Denmark) to >95% purity, and were solubilized in MES buffer at a concentration of 1.25 mM. Next, 20-μl mixtures of *Mtb*-SecB^TA^ protein (10 μM) and SYPRO Orange ( × 10; Invitrogen) were subjected to a temperature gradient from 15 to 90 °C with increments of 0.3 °C. All measurements were performed in triplicate in 96-well plates (Bio-Rad). Thermal transitions were monitored with a real-time PCR CFX96 System (Bio-Rad). The melting points (*T*_m_) were identified by the inflexion points of the curves in relative fluorescence units=*f(T)*. For DSF experiments in the presence of the 13-mer peptides, the final peptide concentrations were chosen to set peptide/protein molar ratios to 12.5:1.0 and 60:1.0 (peptide:chaperone).

### *In vitro* aggregation assay

Unfolded *Mtb*-HigA1 WT, W108A/W137A or Y114A (in 20 mM Tris (pH 7.5), 150 mM NaCl, 1 mM DTT, 20% glycerol and 8 M urea) were diluted to 60 μM in 6.5 M urea and 10 mM DTT at room temperature for 2 h. Proteins were then diluted to 4 μM in PBS and light scattering was measured in a fluorimeter (Fluoromax-4, Horiba Scientific) at 25 °C (*λ*_ex_: 350 nm; *λ*_em_: 355 nm; slits: 1,5 nm, integration time: 1 s, maximal level of stirring). Measurements were carried out in triplicates.

### Cell-free protein synthesis and aggregation assay

Cell-free transcription/translation-coupled *in vitro* assay using the PURE system was carried out according to ref. 17. In brief, DNA of the various constructs (see Supplementary Table 1 for a list of primers) was added at a final concentration of 7.2 ng μl^−1^ to the PURE system, with or without purified *Mtb*-SecB^TA^. Protein synthesis was performed at 37 °C for 60 min with 0.4 μCi μl^−1^ of ^35^S-methionine. An aliquot was withdrawn as the total fraction and the remaining reaction was centrifuged at 21,600*g* for 30 min, and the supernatant fraction was collected. Both fractions were separated on 4–20% Mini-Protean TGX gels (Bio-Rad), visualized using a Typhoon phosphorimager (GE Healthcare) and quantified using Multigauge software (Fuji). Results are presented as the mean and s.d. of triplicate experiments.

### Data availability

The data that support the findings of this study are available from the corresponding author on request.

## Additional information

**How to cite this article:** Bordes, P. *et al*. Chaperone addiction of toxin-antitoxin systems. *Nat. Commun.* **7**, 13339 doi: 10.1038/ncomms13339 (2016).

**Publisher's note:** Springer Nature remains neutral with regard to jurisdictional claims in published maps and institutional affiliations.

## Supplementary Material

Supplementary Information Supplementary Figures 1-8 and Supplementary Table 1.
