# Supplementary material for: Chaperone addiction of toxin–antitoxin systems
Source: Nat Commun. 2016 Nov 9;7:13339. doi: 10.1038/ncomms13339 (PMC5105189; doi:10.1038/ncomms13339)
Supplement: Supplementary Information — Supplementary Figures 1-8 and Supplementary Table 1. [file ncomms13339-s1.pdf]

SUPPLEMENTARY INFORMATION

SUPPLEMENTARY FIGURES

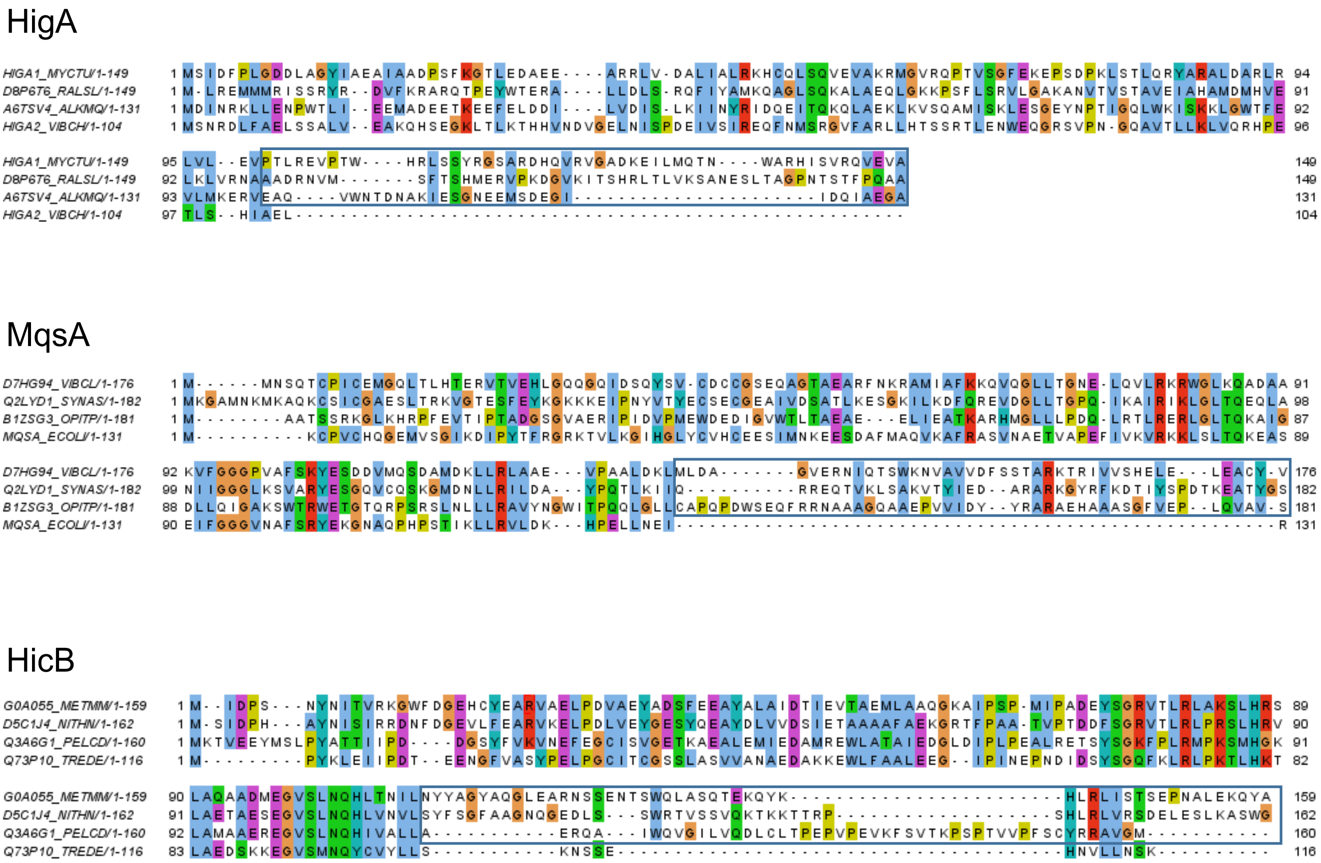

Supplementary Figure 1: Sequence comparison of TAC antitoxins with their prototypical two-component antitoxin homologue (related to Fig. 1 and 2). Protein sequences were aligned using ClustalW. For each family of antitoxin generally found associated with a SecB-like chaperone (HigA, MqsA and HicB), three TAC antitoxins were aligned with a prototypical example of their respective two-component antitoxin homologues. Antitoxin sequences (with UniProt database IDs) used are *Mycobacterium tuberculosis* (strain H37Rv), HIGA1_MYCTU; *Ralstonia solanacearum* (strain CFBP2957), D8P6T6_RALSL; *Alkaliphilus metalliredigens* (strain QYMF), A6TSV4_ALKMQ; *Vibrio cholerae serotype O1* (strain ATCC39315), HIGA2_VIBCH; *Vibrio cholerae* (strain RC385), D7HG94_VIBCL; *Syntrophus aciditrophicus* (strain SB), Q2LYD1_SYNAS; *Opitutus terrae* (strain DSM11246), B1ZSG3_OPITP; *Escherichia coli* (strain K12), MQSA_ECOLI; *Methylomonas methanica* (strain MC09), G0A055_METMM; *Nitrosococcus halophilus* (strain Nc4), D5C1J4_NITHN; *Pelobacter carbinolicus* (strain DSM2380), Q3A6G1_PELCD; *Treponema denticola* (strain DSM14222), Q73P10_TREDE. The C-terminal extensions found in TAC antitoxins are boxed in blue.

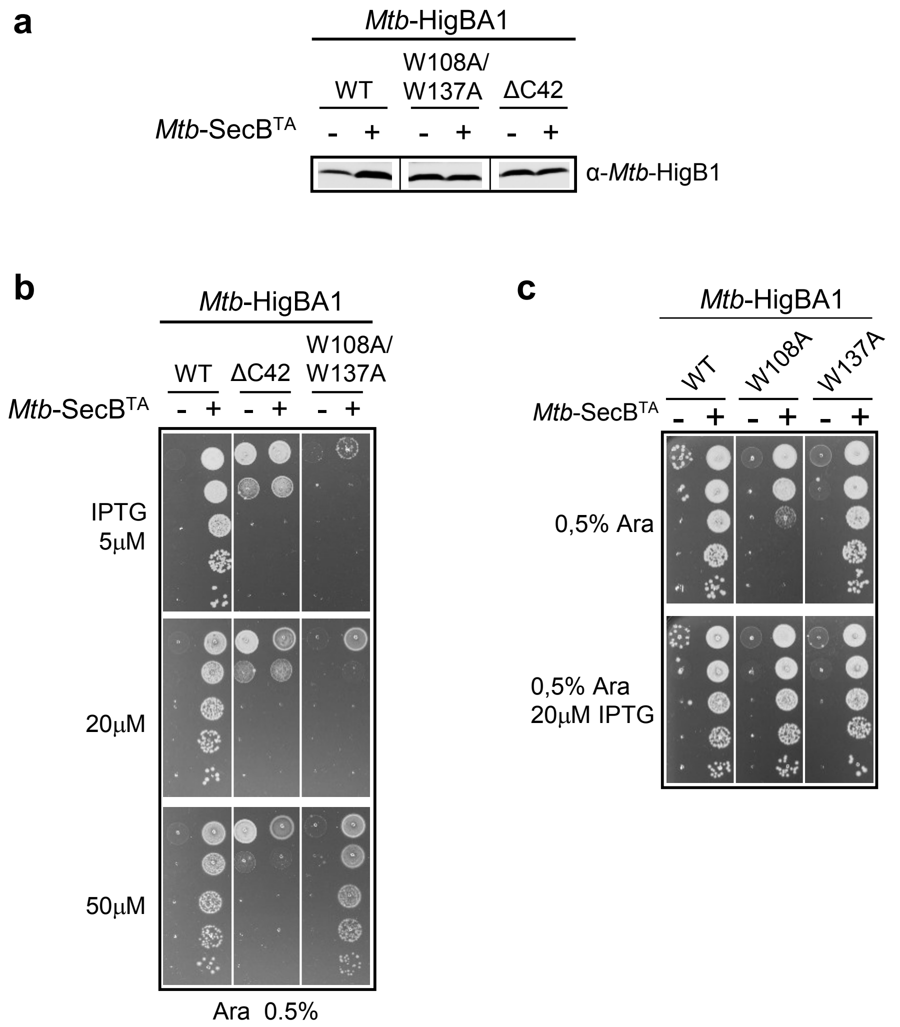

Supplementary Figure 2: Effect of mutations of the C-terminal extension of *Mtb*-HigA1 on the suppression of *Mtb*-HigB1 toxicity (related to Fig. 1). **a,** Steady state expression level of *Mtb*-HigB1 toxin is not affected by mutations in *Mtb*-HigA1 antitoxin in *E. coli*. Double transformants of strainW3110 *ΔsecB* were grown to midlog phase and expression of both *Mtb-higBA1* and *Mtb-SecB^{TA}* was induced for 1h with 0,1% arabinose and 50μM IPTG, respectively. Whole-cell extracts were separated by SDS-PAGE and analyzed by western blot using anti-*Mtb*-HigB1 antibodies. Full blots for (a) are shown in Supplementary Fig. 6. **b,** Strain W3110 Δ*secB* containing the plasmid pSE-vector (-) or pSE-*Mtb*-SecB^{TA} was transformed with pK6-*Mtb*-HigBA1 wild type, ΔC42 or W108A/W137A. Double transformants were grown in LB–ampicillin–kanamycin to midlog phase, serially diluted, and spotted on LB–ampicillin–kanamycin agar plates with arabinose and IPTG as indicated. Plates were incubated at 37°C overnight. **c,** Strain W3110 Δ*secB* containing the plasmid pSE-vector (-) or pSE-*Mtb*-SecB^{TA} was transformed with pK6-*Mtb*-HigBA1 wild type, or with the single W108A or W137A mutants. Double transformants were grown in LB–ampicillin–kanamycin to midlog phase, serially diluted, and spotted on LB–ampicillin–kanamycin agar plates with arabinose and IPTG as indicated. Plates were incubated at 37°C overnight.

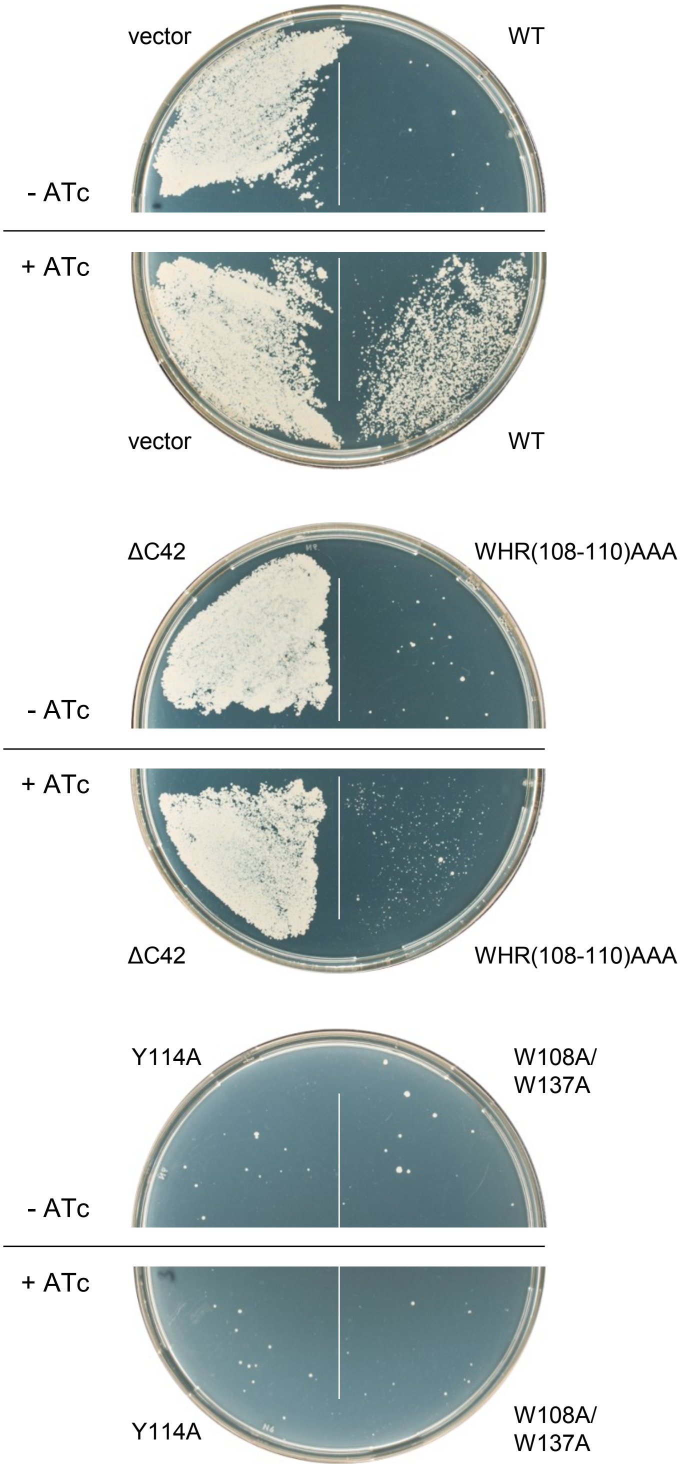

Supplementary Figure 3: Effect of the identified mutations on toxin inhibition *in vivo* in *M. smegmatis* (related to Fig. 1 and 3)**.** Plasmid pLAM12 derivatives co-expressing the toxin *Mtb*-HigB1 and the antitoxin *Mtb*-HigA1 either wild type or carrying the ΔC42, W108A/W137A, WHR(108-110)AAA or Y114A mutations under the control of an acetamide inducible promoter were electroporated into the *M. smegmatis* MC^2155 pGMC-Rv1957 strain, which has the *Mtb*-$SecB^{TA}$ chaperone integrated at the L5 phage attachment site under the control of an anhydrotetracycline inducible promoter. After 4 hours of incubation at 37°C, 1/100 of the transformations were directly plated on LB agar plates containing Streptomycin (25µg/mL) and Kanamycin (5µg/mL) with and without 20ng/mL anhydrotetracycline (ATc) to induce or not the expression of the chaperone. Plates were incubated at 37°C for 48h.

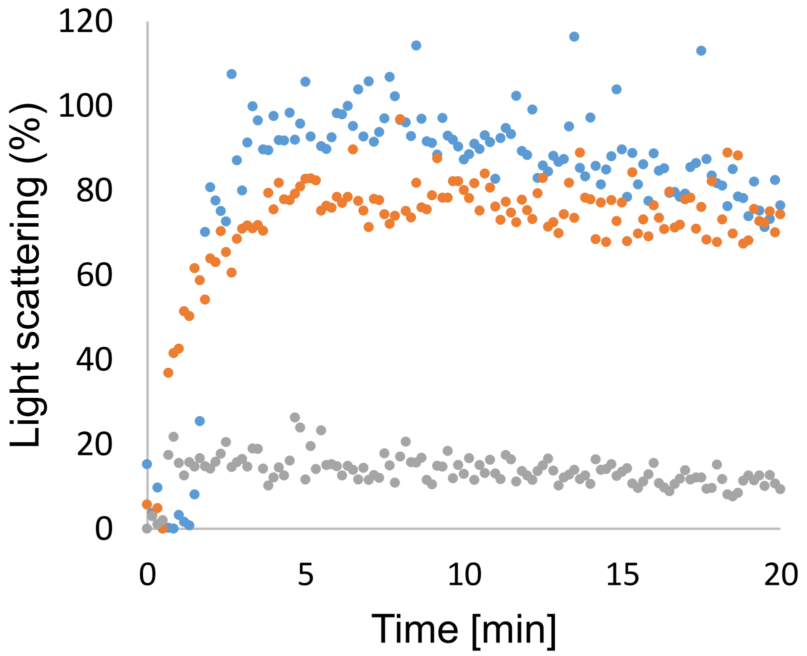

Supplementary Figure 4: Effect of Y114A mutation on *Mtb*-HigA1 antitoxin aggregation (related to Fig. 3). Measurement of light scattered by 1 µM urea-DTT denatured *Mtb*-HigA1 wild type (blue), Mtb-HigA1^{Y114A} (orange) and Mtb-HigA1$^{W108A/W137A}$ (gray) after dilution in PBS at 25°C. The graph is a representative result of three independent measurements.

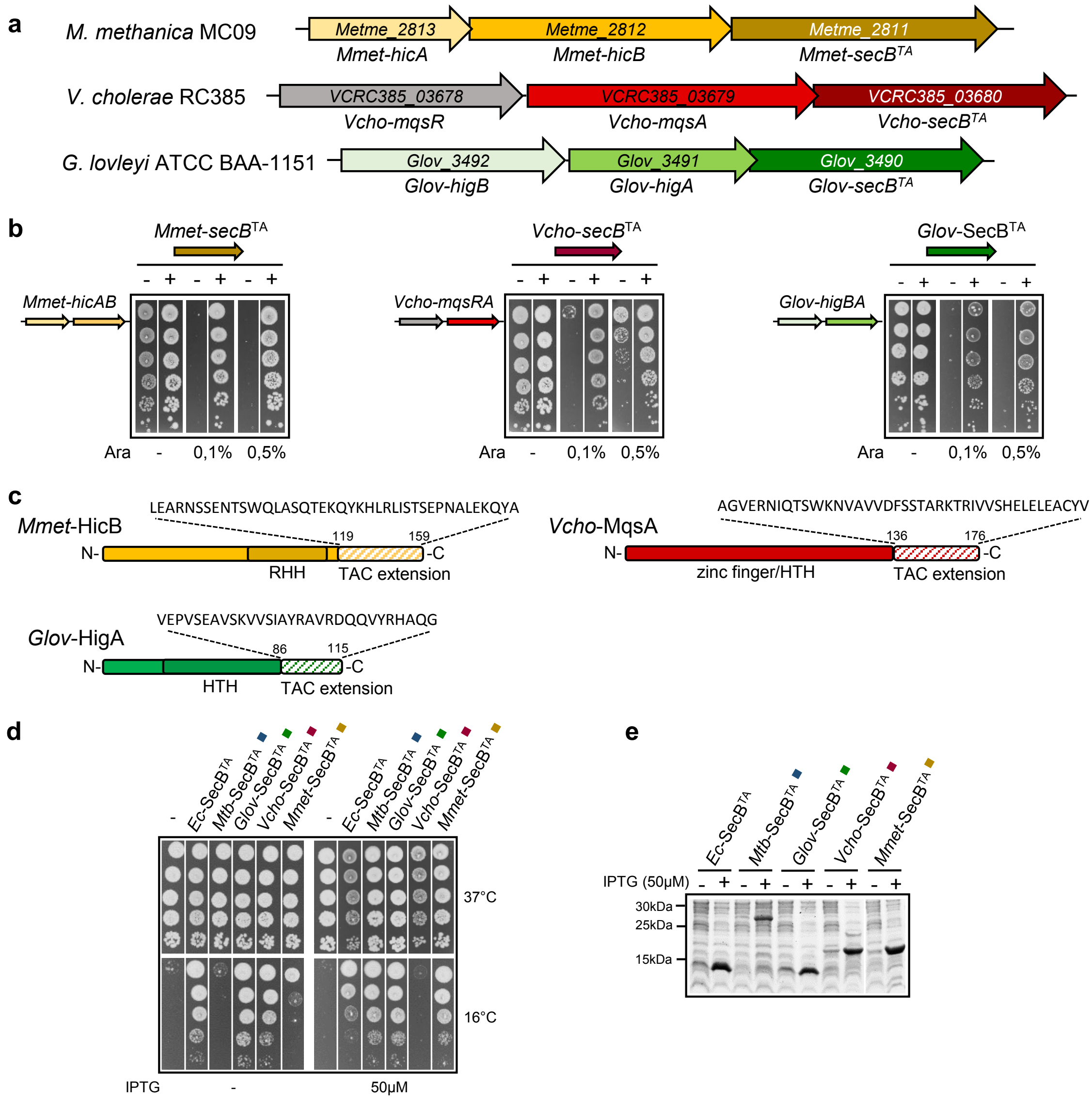

Supplementary Figure 5: ***M. methanica*, *V. cholera, G. lovleyi* TAC systems** (related to Fig. 4). **a,** Schematic representation of the new TAC systems of *M. methanica* MC09, *V. cholerae* RC385 and *G. lovleyi* ATCC BAA-1151 strains. **b,** The operons from *M. methanica*, *V. cholerae* and *G. lovleyi* are *bona fide* TAC in *E. coli*. Strain W3110 Δ*secB* was co-transformed with pSE vector (-) or pSE-*Mmet*-SecB^{TA} and pK6-*Mmet*-HicAB (left), or pSE-*Vcho*-SecB^{TA} and pK6-*Vcho*-MqsRA (center), or pSE-*Glov*-SecB^{TA} and pK6-*Glov*-HigBA (right), on LB–ampicillin–kanamycin agar plates containing 0.2% glucose at 37°C. Double transformants were grown in LB–ampicillin–kanamycin to midlog phase, serially diluted, and spotted on LB–ampicillin–kanamycin agar plates with IPTG and arabinose inducers as indicated. Plates were incubated at 37°C overnight. **c,** Schematic representation of *M. methanica* (left), *V. cholerae* (center) and *G. lovleyi* (right) antitoxins. The TAC specific C-terminal extensions (hachures) and their amino acid sequences are shown. **d,** *M. methanica*, *V. cholerae, G. lovleyi* SecB-like chaperones replace SecB in *E. coli*. Transformants of W3110 Δ*secB* containing pSE derivatives harboring SecB-like chaperones were spotted on LB-ampicillin with or without IPTG, and incubated at the indicated temperature. **e,** Expression of SecB^{TA} chaperones. Transformants of W3110 Δ*secB* containing pSE-SecB-like chaperone derivatives were grown to midlog phase, IPTG (50µM) was added for 2 hours. Whole-cell extracts were separated by SDS-PAGE and stained with Coomassie.

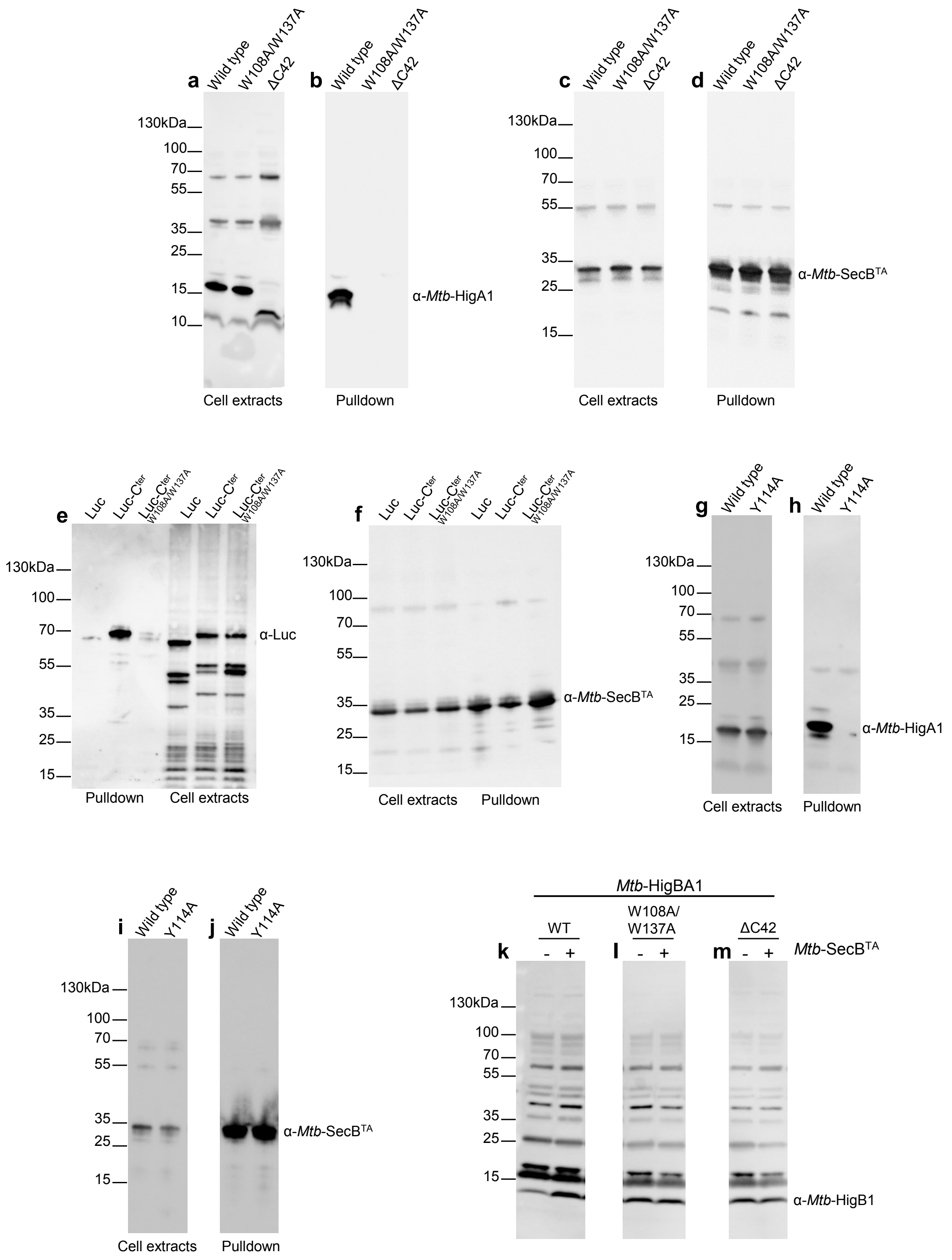

Supplementary Figure 6: Full western blots of *Mtb*-HigA1, *Mtb*-SecB^{TA} and luciferase. *In vivo* pulldowns of His-tagged *Mtb*-SecB^{TA} and the different *Mtb*-HigA1 (wild type)*, Mtb*-HigA1$^{W108A/W137A}$ (W108A/W137A) and *Mtb*-HigA1$^{\Delta C42}$ (ΔC42) proteins and whole cell extracts revealed using anti-*Mtb*-HigA1 (a and b) or with anti-*Mtb*-SecB^{TA} antibodies (c and d). Blots in (a, b, c, d) are the same as in Fig. 1b. *In vivo* pulldowns of His-tagged *Mtb*-SecB^{TA} and the pK6-Luc constructs and whole cell extracts were revealed with anti-Luciferase (e) or anti-*Mtb*-SecB^{TA} (f) antibodies. Blots in (e, f) are the same as in Fig. 1c. *In vivo* pulldown of His-tagged *Mtb*-SecB^{TA} and *Mtb*-HigA1 (wild type) or *Mtb*-HigA1^{Y114A} (Y114) proteins, and whole cell extracts were revealed using anti-*Mtb*-HigA1 (g and h) or anti-*Mtb*-SecB^{TA} antibodies (i and j). Blots in (g, h, i, j) are the same as in Fig. 3e. *Mtb*-HigB1 steady state expression levels in the presence of *Mtb*-HigA1 (k)*, Mtb*-HigA1$^{W108A/W137A}$ (l) or *Mtb*-HigA1$^{\Delta C42}$ (m). Blots in (k, l, m) are the same as in Supplementary Fig. 2a.

a

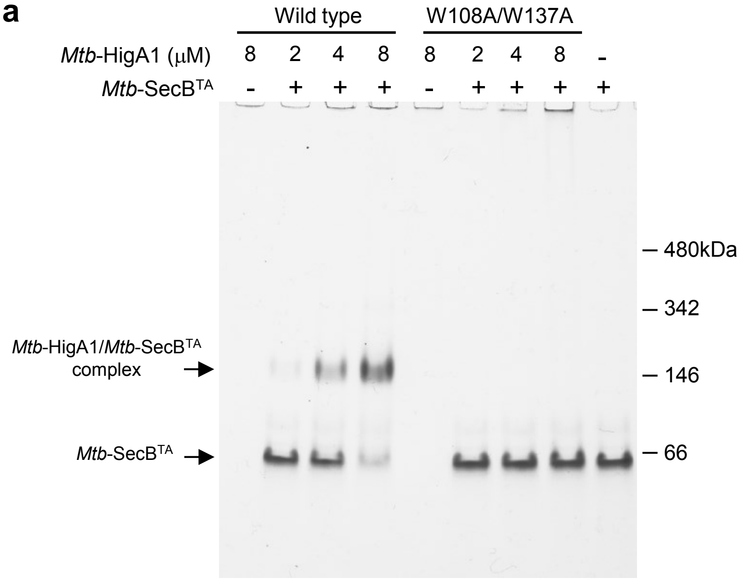

b

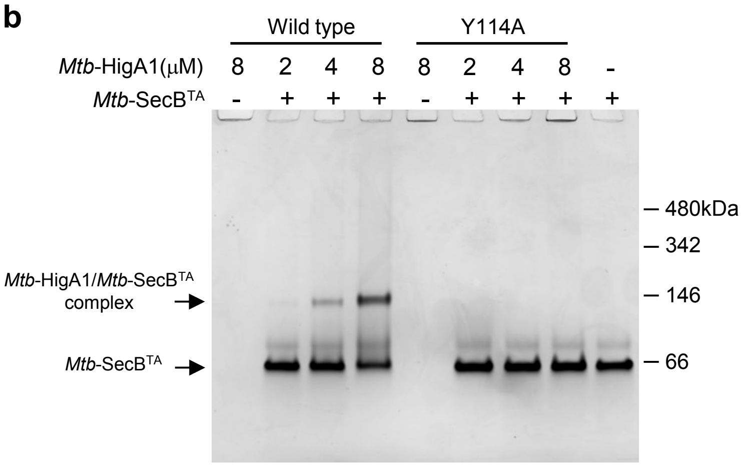

Supplementary Figure 7: Full native gels of *Mtb*-HigA1 wild type and *Mtb*-HigA1$^{W108A/W137A}$ with or without *Mtb*-SecB^{TA} chaperone (a) from Fig 1d, and *Mtb*-HigA1 wild type with *Mtb*-HigA1^{Y114A} (b) from Fig 3f.

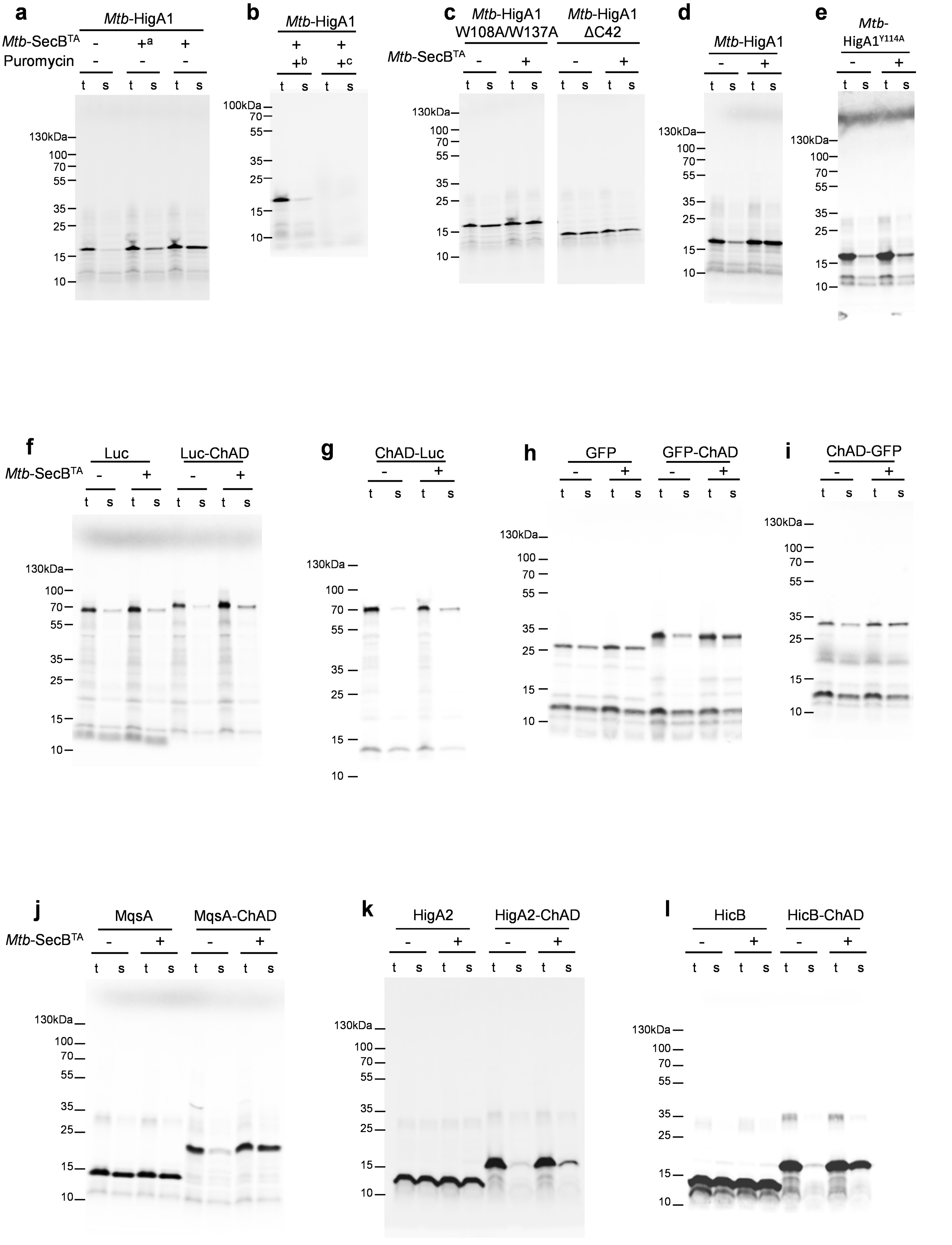

Supplementary Figure 8: Full phosphorimager images of *Mtb*-HigA1, luciferase, GFP, *Eco*-MqsA, *Vcho*-HigA2 and *Tde*-HicB. Phosphorimager images of *Mtb*-HigA1 wild type, *Mtb*-HigA1$^{\Delta C42}$, *Mtb*-HigA1$^{W108A/W137A}$, and *Mtb*-HigA1^{Y114A} mutant derivatives expressed in a cell-free translation system. (a, b and c) are uncropped images from Fig 2a and (d and e) from Fig 3g. Phosphorimager images of Luc (f and g) and GFP (h and i), expressed in a cell-free translation system with or without grafted ChAD sequence. (f and g) are uncropped images from Fig 2c and (h and i) from Fig 3d. Phosphorimager images of *Eco*-MqsA (j), *Vcho*-HigA2 (k) and *Tde*-HicB (l) expressed in a cell-free translation system with or without grafted ChAD sequence are uncropped images from Fig 5b.

Supplementary Table 1: Specific primers for the PURE transcription/translation system

Translation product	Primers (5' to 3')
Mtb-HigA	FPUREHigAPT7:gcgaattaatacgactcactatagggcttaagtataaggaggaaaaaatatgagcattgacttccctttgggtgac and RPUREHigATT7:aaacccctccgtttagagaggggttatgctagtcatgccacctcaacctgccgaacc
Luc	FPURELuciferasePT7:gcgaattaatacgactcactatagggcttaagtataaggaggaaaaaatatggaagacgccaaaaacataaagaaagg and RPURELuciferaseTT7:aaacccctccgtttagagaggggttatgctagttacacggcgatctttccgcccttcttgg
Luc-*Mtb*-ChAD	F PURELuciferasePT7 and R PUREHigATT7
Mtb-ChAD-Luc	FPUREC1PT7:gcgaattaatacgactcactatagggcttaagtataaggaggaaaaaatatggaagtgcctacgtggcatcggctc and RPURELuciferaseTT7
Eco-MqsA	FPUREMqsAPT7:gcgaattaatacgactcactatagggcttaagtataaggaggaaaaaatatgaaatgtccggtttgccaccaggg and RPUREMqsATT7:aaacccctccgtttagagaggggttatgctagttaacggatttcattcaatagttctggatg
Eco-MqsA-*Mtb*-ChAD	FPUREMqsAPT7 and RPUREHigATT7
GFP	FPUREGfpPT7:gcgaattaatacgactcactatagggcttaagtataaggaggaaaaaatatggagtaaaggagaagaacttttcactg and RPUREGfpTT7:aaacccctccgtttagagaggggttatgctagttatttgtagagctcatccatgccatgtg
GFP-*Mtb*-ChAD	FPUREGfpPT7 and RPUREHigATT7
Mtb-ChAD-GFP	FPUREC1PT7 and RPUREGfpTT7
Vcho-higA2	FPUREHigA2VcPT7:gcgaattaatacgactcactatagggcttaagtataaggaggaaaaaatatgagcaatcgtgatttatttgcag and RPUREHigA2VcTT7:aaacccctccgtttagagaggggttatgctagttatagctcggctatgtgtgacaacgtttc
Vcho-HigA2-*Mtb*-ChAD	FPUREHigA2VcPT7 and RPUREHigATT7
Tde-HicB	FPUREHicBTDE0481PT7:gcgaattaatacgactcactatagggcttaagtataaggaggaaaaaatatgcaaataaaggattatttaaatttac and RPUREHicBTDE0481TT7: aaacccctccgtttagagaggggttatgctagttaagaaagtctataaagaacgtattg
Tde-HicB-*Mtb*-ChAD	FPUREHicBTDE0481PT7 and RPUREHigATT7
